# Quantifying Oxidation of Cellulose-Associated Glucuronoxylan by Two Lytic Polysaccharide Monooxygenases from Neurospora crassa

**DOI:** 10.1128/AEM.01652-21

**Published:** 2021-11-24

**Authors:** Olav A. Hegnar, Heidi Østby, Dejan M. Petrović, Lisbeth Olsson, Anikó Várnai, Vincent G. H. Eijsink

**Affiliations:** a Norwegian University of Life Sciences, Faculty of Chemistry, Biotechnology and Food Science, Ås, Norway; b Department of Biology and Biological Engineering, Division of Industrial Biotechnology, Chalmers University of Technology, Gothenburg, Sweden; c Wallenberg Wood Science Center, Chalmers University of Technology, Gothenburg, Sweden; Nanjing Agricultural University

**Keywords:** lytic polysaccharide monooxygenases, LPMO, lignocellulose, *Neurospora crassa*, xylan, hemicellulose, glucuronoxylan

## Abstract

Family AA9 lytic polysaccharide monooxygenases (LPMOs) are abundant in fungi, where they catalyze oxidative depolymerization of recalcitrant plant biomass. These AA9 LPMOs cleave cellulose and some also act on hemicelluloses, primarily other (substituted) β-(1→4)-glucans. Oxidative cleavage of xylan has been shown for only a few AA9 LPMOs, and it remains unclear whether this activity is a minor side reaction or primary function. Here, we show that Neurospora crassa LPMO9F (*Nc*LPMO9F) and the phylogenetically related, hitherto uncharacterized *Nc*LPMO9L from N. crassa are active on both cellulose and cellulose-associated glucuronoxylan but not on glucuronoxylan alone. A newly developed method for simultaneous quantification of xylan-derived and cellulose-derived oxidized products showed that *Nc*LPMO9F preferentially cleaves xylan when acting on a cellulose–beechwood glucuronoxylan mixture, yielding about three times more xylan-derived than cellulose-derived oxidized products. Interestingly, under similar conditions, *Nc*LPMO9L and the previously characterized *Mc*LPMO9H, from *Malbranchea cinnamomea*, showed different xylan-to-cellulose preferences, giving oxidized product ratios of about 0.5:1 and 1:1, respectively, indicative of functional variation among xylan-active LPMOs. Phylogenetic and structural analysis of xylan-active AA9 LPMOs led to the identification of characteristic structural features, including unique features that do not occur in phylogenetically remote AA9 LPMOs, such as four AA9 LPMOs whose lack of activity toward glucuronoxylan was demonstrated in the present study. Taken together, the results provide a path toward discovery of additional xylan-active LPMOs and show that the huge family of AA9 LPMOs has members that preferentially act on xylan. These findings shed new light on the biological role and industrial potential of these fascinating enzymes.

**IMPORTANCE** Plant cell wall polysaccharides are highly resilient to depolymerization by hydrolytic enzymes, partly due to cellulose chains being tightly packed in microfibrils that are covered by hemicelluloses. Lytic polysaccharide monooxygenases (LPMOs) seem well suited to attack these resilient copolymeric structures, but the occurrence and importance of hemicellulolytic activity among LPMOs remain unclear. Here, we show that certain AA9 LPMOs preferentially cleave xylan when acting on a cellulose–glucuronoxylan mixture, and that this ability is the result of protein evolution that has resulted in a clade of AA9 LPMOs with specific structural features. Our findings strengthen the notion that the vast arsenal of AA9 LPMOs in certain fungal species provides functional versatility and that AA9 LPMOs may have evolved to promote oxidative depolymerization of a wide variety of recalcitrant, copolymeric plant polysaccharide structures. These findings have implications for understanding the biological roles and industrial potential of LPMOs.

## INTRODUCTION

In nature, decomposition of plant biomass is primarily performed by fungi. The degradation of plant cell walls requires a large suite of enzymes that work in concert to hydrolyze and oxidize its major polymeric components: cellulose, hemicelluloses, and lignin (1). In fungi, the major secreted enzymes that act on plant cell wall polysaccharides are glycoside hydrolases (GHs), carbohydrate esterases (CEs), and lytic polysaccharide monooxygenases (LPMOs) (2–7). Dikaryotic fungi carry genes encoding LPMOs from five currently recognized LPMO families, namely, AA9, AA11, AA13, AA14, and AA16, that act on various crystalline and amorphous polysaccharides, primarily cellulose and chitin (8). LPMOs are mono-copper enzymes that oxidize chitin or cellulose by hydroxylating either the C-1 or C-4 position of the scissile glycosidic bond, which leads to spontaneous bond cleavage (9–13). LPMOs were originally considered monooxygenases, using O_2_ as a cosubstrate (9, 14), but recent work indicates that LPMOs are efficient peroxygenases, using H_2_O_2_ as a cosubstrate (15–19).

Family AA9 LPMOs are cellulose-active enzymes, some of which can also cleave hemicelluloses containing β-(1→4)-linked glucose units in the polysaccharide backbone, like glucomannan and xyloglucan (20). In addition, oxidative cleavage of xylan has been convincingly demonstrated for two AA9 LPMOs, *Mt*LPMO9A from *Myceliophthora thermophila* (21), originally named *Mt*LPMO9E by Berka et al. (22), and *Mc*LPMO9H from *Malbranchea cinnamomea* (23), both of which are monomodular and (primarily) C-1-oxidizing enzymes, sharing 55.6% sequence identity. These two enzymes produce oxidized xylo-oligomers when incubated with cellulose–glucuronoxylan copolymeric mixtures but are inactive toward soluble xylan alone. The inactivity on soluble xylan is likely due to the 3-fold screw conformation that this polymer has in solution, which is flexible and nonuniform, whereas xylan adopts a 2-fold screw conformation when associated with cellulose, leading to a more rigid and “crystalline” structure (24). It is well known that acetylated, arabinosylated, and/or glucuronylated xylans extracted from various sources, including crops, hardwood, and softwood, interact with cellulose surfaces to various extents (25, 26). It has been shown that glucuronoxylans with even pattern substitution, including acetylglucuronoxylan from *Arabidopsis* (27) and glucuronoarabinoxylan from spruce (28), adapt 2-fold screw conformation upon adsorption to cellulose in plant cell walls.

In a landmark study from 2015, Frommhagen et al. (21) showed production of oxidized xylo-oligomers upon incubation of *Mt*LPMO9A with a mixture of birchwood glucuronoxylan or oat spelt arabinoxylan and regenerated amorphous cellulose. These LPMO products were detected using high-performance anion-exchange chromatography with pulsed amperometric detection (HPAEC-PAD) and matrix-assisted laser desorption-ionization time-of-flight mass spectrometry (MALDI-TOF MS). A similar approach was taken by Hüttner et al. (23) studying *Mc*LPMO9H, where reactions were performed with mixtures of phosphoric acid swollen cellulose (PASC) and birchwood 4-*O*-methylglucuronoxylan. In this case, a wide variety of oxidized xylan products were detected by MALDI-TOF MS, but HPAEC-PAD detection was not described. Additionally, *Ls*LPMO9A from *Lentinus similis* has been suggested by Simmons et al. to act on birchwood glucuronoxylan (29), as it produces soluble native xylo-oligomers in a reductant-dependent manner, although the authors were unable to detect any oxidized xylo-oligomers.

In 2018, a novel xylan-active LPMO family, AA14, was discovered (30), with, until now, only two characterized members, both from the white-rot fungus *Pycnoporus coccineus*. In contrast to the xylan-active AA9 LPMOs, these enzymes are not active on cellulose but are thought to cleave highly refractory xylan that is grafted onto cellulose. Of possible products, only xylotrionic acid (Xyl2Xyl1A) was detected, by mass spectrometry only (30). More recently, it was shown that an AA14 enhances the release of native xylo-oligosaccharides from xylan-rich cellulose fibers by a xylobiohydrolase (31).

The above-mentioned discovery of LPMO activity on cellulose–xylan complexes provides a glimpse of functional diversity among LPMOs that may be needed to degrade different copolymeric structures occurring in plant cell walls, and that may explain why some biomass-degrading fungi carry up to about 50 LPMO genes. Still, despite the above-mentioned and other findings (e.g., by Petrović et al. [32]), the functional implications of LPMO multiplicity remain poorly understood. Furthermore, not all functionally characterized LPMOs have been characterized to the same extent, which means that certain activities may have remained undetected. For example, considering the abundance of xylan–cellulose copolymeric structures in plant cell walls, one would perhaps expect a greater occurrence, and more in-depth characterization, of xylan-active LPMOs.

The genome of Neurospora crassa, an ascomycete bread mold found on decaying leaves in nature, encodes 14 AA9 LPMOs (33) but no AA14 LPMOs, which are primarily found in *Basidiomycetes* (30). At the time of writing, 9 of the 14 AA9 LPMOs in N. crassa had been functionally characterized to various extents (32, 34, 35): *Nc*LPMO9A (*gh61-1*, NCU02240), -9B (*gh61-2*, NCU07760), -9C (*gh61-3*, NCU02916), -9D (*gh61-4*, NCU01050), -9E (*gh61-5*, NCU08760), -9F (*gh61-6*, NCU03328), -9G (*gh61-7*, NCU00836), -9J (*gh61-10*, NCU01867), and -9M (*gh61-13*, NCU07898) (32, 34, 35), while the other five AA9 LPMOs, *Nc*LPMO9H (*gh61-8*, NCU03000), -9I (*gh61-9*, NCU05969), -9K (*gh61-11*, NCU07520), -9L (*gh61-12*, NCU02344), and -9N (*gh61-14*, NCU07974), await functional characterization. N. crassa currently is the best-characterized fungus in terms of its LPMO repertoire. All characterized N. crassa LPMOs are active on cellulose, four are C-1 oxidizing (*Nc*LPMO9E, -9F, -9G, and -9J), three are C-4 oxidizing (*Nc*LPMO9A, -9C, and -9D), two are C-1/C-4 oxidizing (*Nc*LPMO9B and -9M), and six of them carry CBM1 domains (three C-1 oxidizing, *Nc*LPMO9E, -9G, and -9J; two C-4 oxidizing, *Nc*LPMO9A and -9C; one C-1/C-4 oxidizing, *Nc*LPMO9B). Among these, *Nc*LPMO9F, a monomodular LPMO that oxidizes cellulose at the C-1 position, is one of the best-studied AA9 LPMOs. Its activity on cellulose was demonstrated in 2012 (35), and its crystal structure was solved in 2015 (36). In support of the idea that these many LPMOs have different functional roles, it is well established that fungal LPMO genes are differentially expressed both temporally and in response to different substrates, which is also true for N. crassa LPMOs (37–39).

**FIG 1:**
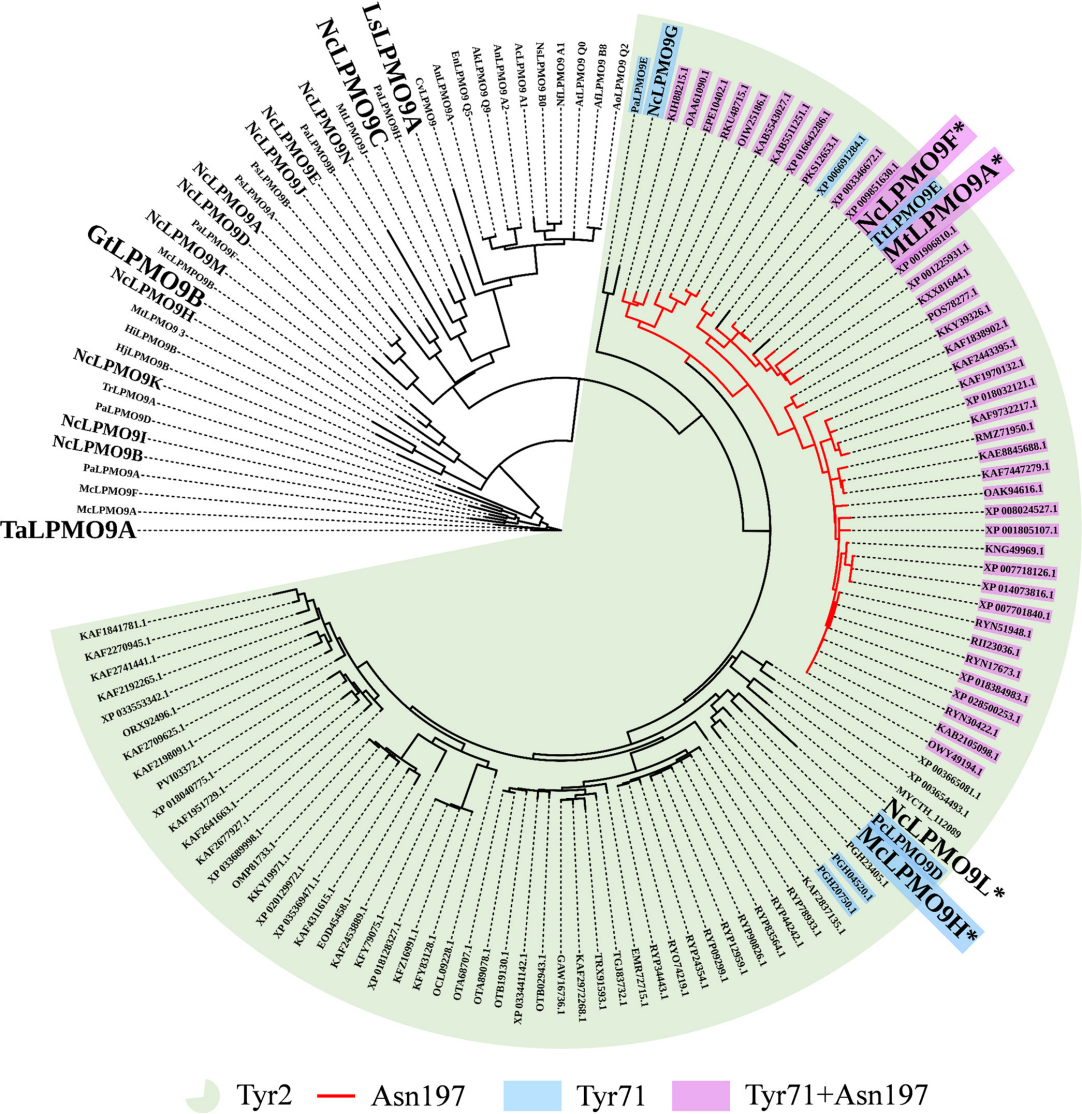
Phylogenetic distance tree of AA9 LPMOs. Multiple-sequence analysis of 34 functionally characterized AA9 LPMOs, all 14 N. crassa AA9 LPMOs, *Mc*LPMO9H, and 91 uncharacterized AA9 LPMOs that are most similar to *Nc*LPMO9F and *Mc*LPMO9H was performed using Expresso (T-Coffee), with subsequent phylogenetic analysis performed with ProtTest 3.4. The lettering size for LPMO names is the following: large, LPMOs that were used in this study plus known xylan-active LPMOs *Mc*LPMO9H and *Mt*LPMO9A; medium, all *Nc*LPMO9s (except 9F, 9L, and 9C, which are large) and previously characterized LPMOs that are discussed in the text; small, all other LPMOs. The names of LPMOs with demonstrated xylanolytic activity are marked by an asterisk. The various colors indicate sequence characteristics, as indicated; see the text for further discussion.

So far, research on AA9 LPMOs has mainly been focusing on cellulose oxidation, while activity toward hemicellulosic substrates, particularly xylans, has been described less frequently. Furthermore, hemicellulolytic activities may have been overlooked because of the use of suboptimal reaction conditions, which may lead to rapid enzyme inactivation and low product levels (40). Strikingly, phylogenetic analysis (Fig. 1; see also Fig. S1 in the supplemental material) showed that the two AA9 LPMOs with clear xylanolytic activity, *Mt*LPMO9A and *Mc*LPMO9H, group together with several well-characterized (C-1-oxidizing) AA9s, including *Nc*LPMO9F from N. crassa (35, 36), for which activity on xylan has not yet been addressed or demonstrated. Another closely related LPMO is the hitherto uncharacterized N. crassa LPMO *Nc*LPMO9L. These four LPMOs belong to a distinct cluster, as is also visible in the analysis of Laurent et al., who classified AA9 LPMOs based on the sequences of five active-site segments (Seg1 to Seg5), placing these LPMOs in a group with relatively short Seg1 and Seg2 segments (41).

Motivated by these phylogenetic observations, we set out to determine if activity on xylan is prevalent among LPMOs that are phylogenetically close to *Mt*LPMO9A and *Mc*LPMO9H, and, if so, if it was possible to identify conserved structural determinants related to xylanolytic activity in AA9 LPMOs. We demonstrate previously overlooked xylanolytic activity of *Nc*LPMO9F, which turned out to preferentially oxidize xylan in 4-*O*-methylglucuronoxylan–cellulose mixtures, and we present a quantitative assessment of xylan oxidation by an LPMO. Additionally, we demonstrate xylanolytic activity for the hitherto uncharacterized *Nc*LPMO9L, which was cloned and expressed as part of this study. Finally, we demonstrate that the preference for cellulose versus xylan in glucuronoxylan–cellulose mixtures varies between xylan-active LPMOs.

## RESULTS AND DISCUSSION

### *Nc*LPMO9F and *Nc*LPMO9L oxidize xylan in cellulose–glucuronoxylan mixtures.

In our phylogenetic analyses, *Nc*LPMO9F (UniProt identifier [ID] Q1K4Q1) from N. crassa clustered closest to the xylan-active *Mt*LPMO9A, whereas the previously uncharacterized *Nc*LPMO9L (UniProt ID Q7S411), also from N. crassa, clustered closest to the xylan-active *Mc*LPMO9H (Fig. 1). These four enzymes shared more than 50% identity with each other (see below for a more detailed discussion; also see Fig. S1 in the supplemental material).

**FIG 2:**
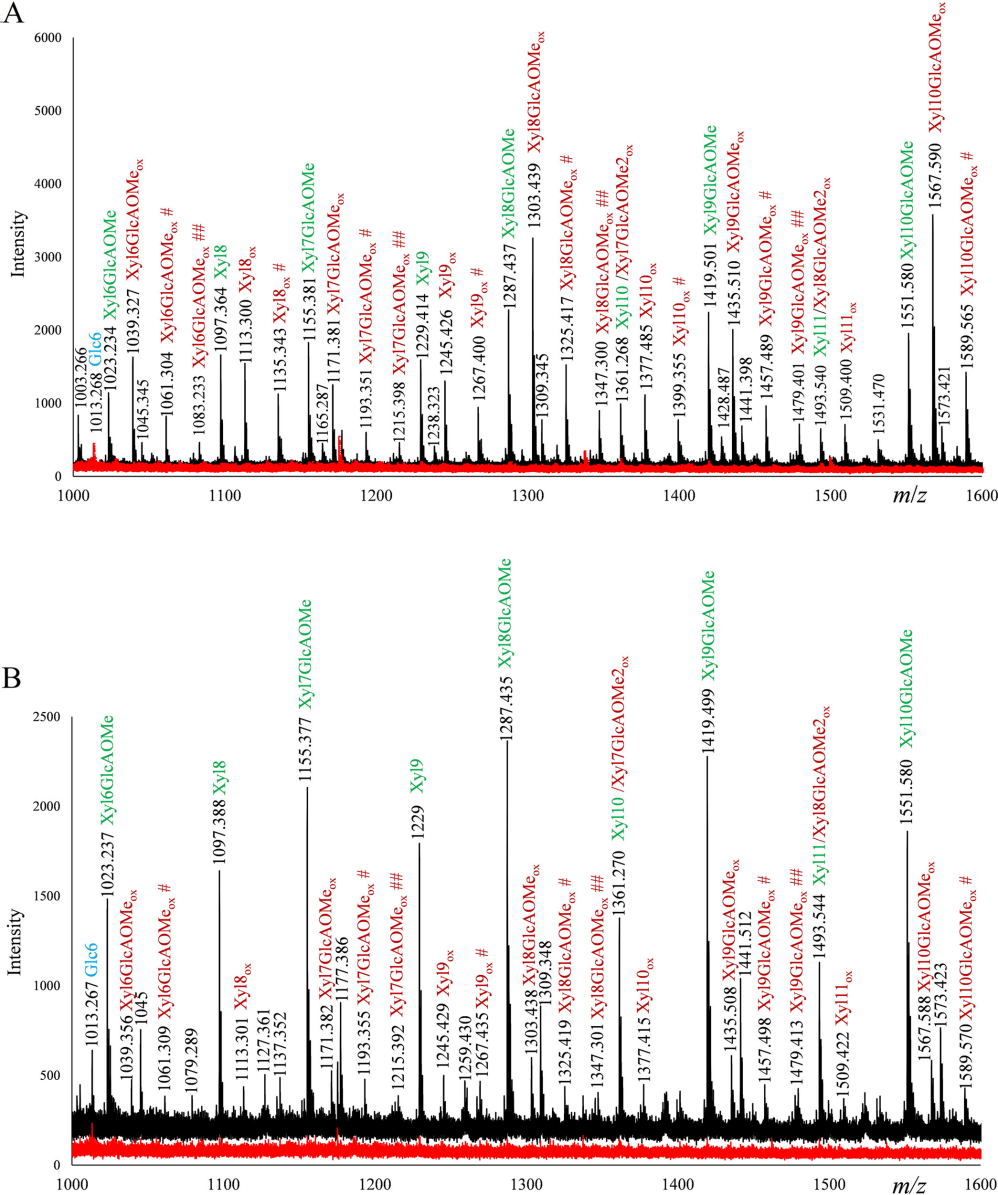
MALDI-TOF MS spectra of products generated by *Nc*LPMO9F and *Nc*LPMO9L in reaction mixtures containing both PASC and BeWX. The reaction mixtures were set up with 1 μM *Nc*LPMO9F (upper) or *Nc*LPMO9L (lower), 0.4% (wt/vol) PASC, and 0.4% (wt/vol) BeWX as the substrate, with (black) or without (red) 1 mM ascorbic acid (AscA) as the reductant, in 50 mM BisTris-HCl buffer, pH 6.0, and incubated at 45°C for 24 h. Reaction mixtures with only PASC generated almost exclusively cellulose-derived products (see Fig. 4 for more details), whereas reactions with only BeWX generated no products (data not shown). All labeled peaks are sodium adducts. Sodium salts (+22 per sodium), which can be formed through binding to GlcAOMe unit(s), and/or the Xyl1A unit are annotated with # or ##, for one or two Na ions, respectively. Oxidized xylan products are labeled in red, while native products are labeled in green; cellulose-derived products are labeled in blue. Note that most cellulose-derived products are not visible in these spectra because of their lower *m/z* values; these products are well visible in the chromatographic analyses shown in other figures. All reactions were performed in triplicate and resulted in similar product profiles.

To test our hypothesis that the phylogenetic clustering and sequence identities of these enzymes translate to similar substrate specificities, such as activity on xylan, we first set up reaction mixtures containing either 0.4% (wt/vol) phosphorous acid swollen cellulose (PASC), 0.4% (wt/vol) beechwood glucuronoxylan (BeWX), or 0.4% (wt/vol) PASC and 0.4% (wt/vol) BeWX in combination. MALDI-TOF MS analysis of product mixtures showed the formation of oxidized xylo-oligosaccharides for both *Nc*LPMO9F and *Nc*LPMO9L (Fig. 2), similar to what has been observed for *Mt*LPMO9A (21) and *Mc*LPMO9H (23). Product mixtures obtained from reaction mixtures containing both PASC and BeWX showed masses corresponding to oxidized nonsubstituted and 4-*O*-methylglucuronylated (i.e., GlcAOMe-substituted) xylo-oligosaccharides, in addition to oxidized cello-oligosaccharides. For *Nc*LPMO9F, the products with the most intense signals include the sodium adducts of native Xyl8GlcAOMe (*m/z *= 1,287), C-1-oxidized Xyl8GlcAOMe (hydrated form; *m/z *= 1,303), native Xyl9GlcAOMe (*m/z *= 1,419), C-1-oxidized Xyl9GlcAOMe (hydrated form; *m/z *= 1,435), and C-1-oxidized Xyl10GlcAOMe (hydrated form; *m/z *= 1,567). Strikingly, xylan-derived products are strongly dominating the product spectrum, which may be taken to suggest that this well-studied cellulose-active LPMO has a preference for xylan, although these differences may also be due to different behaviors of the various products in the MALDI-TOF MS analysis (chromatographic quantification of products is described below). Curiously, for *Nc*LPMO9L, nonoxidized xylan-derived products were more prominent in the MS spectra than oxidized products, whereas the reductant-free control did not show any indications of a background xylanase activity. Both product profiles show signals corresponding to sodium adducts of the sodium salts of oxidized xylo-oligosaccharides that are diagnostic for C-1 oxidations, such as *m/z *= 1,267, which is the sodium adduct of the sodium salt of C-1-oxidized Xyl9, and *m/z *= 1,215, which is the sodium adduct of the double sodium salt of C-1-oxidized Xyl7GlcAOMe.

### Activity on cellulose-associated glucuronoxylan by phylogenetically related LPMOs is detectable with HPAEC-PAD.

**FIG 3:**
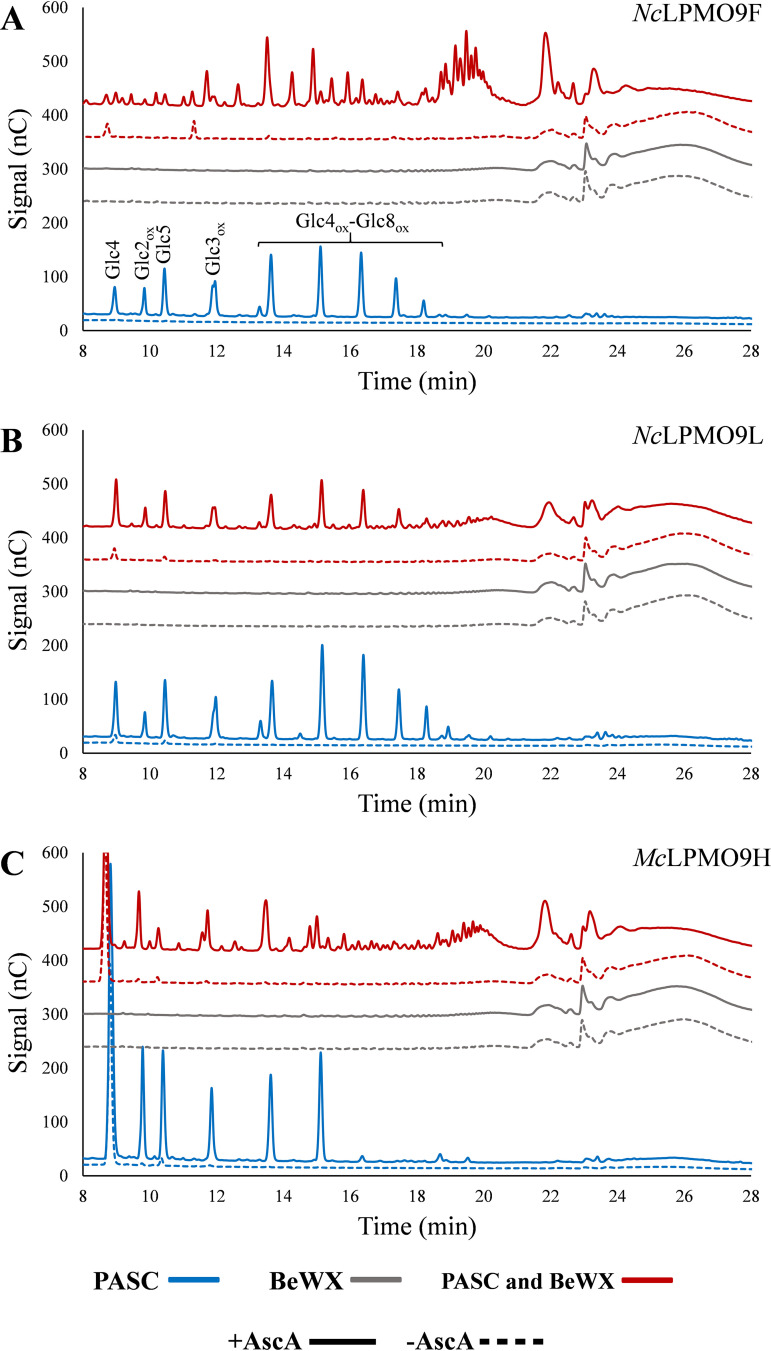
HPAEC-PAD chromatograms of product mixtures from LPMO reactions with PASC, BeWX, or PASC and BeWX. Panels A, B, and C show analyses of reactions with *Nc*LPMO9F, *Nc*LPMO9L, and *Mc*LPMO9H, respectively. All reactions were performed with 1 μM LPMO and either 0.4% PASC, 0.4% BeWX, or 0.4% PASC and 0.4% BeWX, with (solid lines) or without (dashed lines) 1 mM ascorbic acid (AscA), in 50 mM BisTris-HCl buffer, pH 6.0, at 45°C for 24 h. Products in reactions with PASC were native and C-1-oxidized cello-oligomers as indicated in panel A, while reactions with PASC and BeWX showed a mix of native and C-1-oxidized cello- and xylo-oligomers (the xylan-derived products are not annotated). No reductant-dependent products were formed in reactions where BeWX was the only substrate for any of the LPMOs. A more detailed product annotation is provided in Fig. 4. All reactions were performed in triplicate and resulted in identical product profiles.

So far, only Frommhagen et al. have been able to detect (weak) signals for LPMO-generated oxidized xylan-derived oligomers (21). Encouraged by the convincing mass spectra of Fig. 2, we explored the use of HPAEC-PAD for detection of xylan-derived products. HPAEC-PAD analysis of product mixtures obtained from reactions with *Nc*LPMO9F, *Nc*LPMO9L, or *Mc*LPMO9H with a mixture of PASC and BeWX showed peaks for both cellulose- and xylan-derived products for all three LPMOs (Fig. 3). None of these LPMOs were active on BeWX alone, and control reactions without reductant did not yield products (Fig. 3). Assays with PASC alone revealed that the novel LPMO, *Nc*LPMO9L, like *Nc*LPMO9F and *Mc*LPMO9H, oxidizes cellulose at the C-1 position to levels comparable with those obtained with *Nc*LPMO9F (Fig. 3).

Xylanolytic activity of *Nc*LPMO9F, *Nc*LPMO9L, and *Mc*LPMO9H is evident from the plethora of non-cellulose-related peaks that emerge in product mixtures derived from reaction mixtures containing PASC–BeWX mixtures and reductant (Fig. 3). The apparent large product diversity is in accordance with the mass spectrometry data shown in Fig. 2. Strikingly, the ratios between the cellulose- and the xylan-derived products varied a lot for the studied LPMOs, indicating different substrate preferences (Fig. 3). For *Nc*LPMO9F, several of the peaks that did not correspond to the usual cellulose-derived LPMO products had much larger areas than the peaks belonging to cellulose-derived products, which suggests that this LPMO prefers xylan over cellulose, as also suggested by the mass spectrometry data shown in Fig. 2. On the other hand, cellulose-derived products were dominating for *Nc*LPMO9L, while *Mc*LPMO9H showed an intermediate product profile.

**FIG 4:**
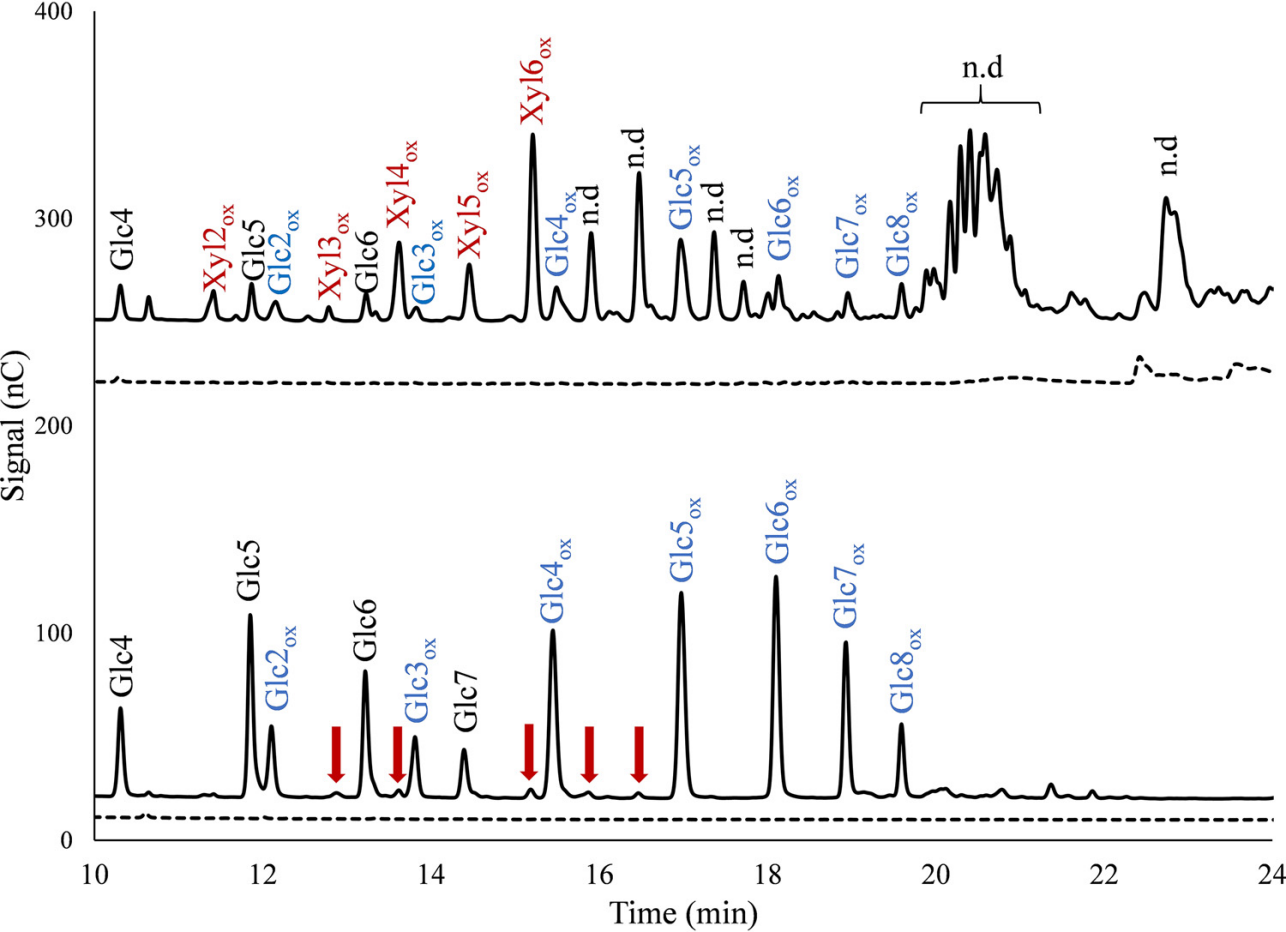
HPAEC-PAD chromatograms of product mixtures generated in reactions of *Nc*LPMO9F with PASC and BeWX (top) or PASC (bottom). Solid chromatograms are for reactions with AscA, while dashed chromatograms are for reactions without AscA. The peaks were annotated using chromatograms of mixtures of native and C-1-oxidized cello- and xylooligosaccharides (DP of 2 to 6). The red arrows indicate oxidized xylo-oligosaccharides resulting from residual xylan in the PASC preparation. Reaction conditions were the same as those for the experiments depicted in Fig. 3. Unannotated putatively xylan-derived products are labeled n.d., for not determined.

To annotate some of the unidentified peaks, we generated C-1-oxidized xylo-oligosaccharide standards (degree of polymerization 2 [DP2] to 6) from linear xylo-oligosaccharides by oxidizing the xylosyl unit at the reducing end to xylonic acid (Xyl1A) using a cellobiose dehydrogenase, *Mt*CDH (see Materials and Methods). This approach allowed the identification of oxidized nonsubstituted xylo-oligosaccharides in the reaction mixture (Fig. 4). The many unidentified peaks are likely unsubstituted oxidized xylan products with a higher degree of polymerization and GlcAOMe-substituted oligomeric xylan products, a notion that is supported by the MALDI-TOF MS data (Fig. 2), which show several oxidized xylo-oligosaccharide products with DP of >6, both nonsubstituted and GlcAOMe substituted. It is noteworthy that product mixtures from reactions with only PASC, *Nc*LPMO9F, and reductant showed small amounts of oxidized xylo-oligosaccharides, likely resulting from oxidative activity on residual xylan in the PASC preparation (Fig. 4). This has also been observed for *Mc*LPMO9H with MALDI-TOF MS analyses (23).

To ensure that the oxidized xylo-oligomers observed did not result from reactions with reactive oxygen species produced in side reactions involving copper, ascorbic acid (AscA), and/or H_2_O_2_ (42), we set up control reaction mixtures where either *Nc*LPMO9F or an equimolar amount of CuSO_4_ was incubated with the substrates in the presence of H_2_O_2_ and AscA (Fig. S2). Indeed, as expected, in reaction mixtures where the LPMO was replaced with CuSO_4_, neither reaction mixtures with BeWX alone nor reaction mixtures with BeWX and PASC generated soluble products. Of note, in reaction mixtures where *Nc*LPMO9F was incubated with BeWX and PASC, we observed significant inhibition of LPMO activity in the reaction with 200 μM H_2_O_2_ (Fig. S2), which is common when LPMO reactions are exposed to higher H_2_O_2_ concentrations. On the other hand, the reaction with 50 μM H_2_O_2_ yielded a product profile similar to that shown in Fig. 3.

Additional reactions were performed with the C-4-oxidizing LPMOs *Nc*LPMO9C and *Ls*LPMO9A, both of which have been shown to cleave oligosaccharides and hemicelluloses with a β-(1→4)-linked glucan backbone (20, 29), the C-1/C-4-oxidizing LPMOs *Ta*LPMO9A from *Thermoascus aurantiacus* and *Gt*LPMO9B from *Gloeophyllum trabeum*, both of which are active on xyloglucan (43, 44), and the C-1-oxidizing cellulose-active bacterial AA10 LPMO CelS2 (*Sc*LPMO10C) from Streptomyces coelicolor (45). For these LPMOs, we were unable to detect xylan-derived products in reactions with the PASC–BeWX mixture, either by HPAEC-PAD or MALDI-TOF MS (data not shown). Of note, weak xylanolytic activity has previously been suggested for *Ls*LPMO9A based on MS signals only (29).

### Quantitative comparison of cellulose and glucuronoxylan oxidation by xylan-active LPMOs.

**FIG 5:**
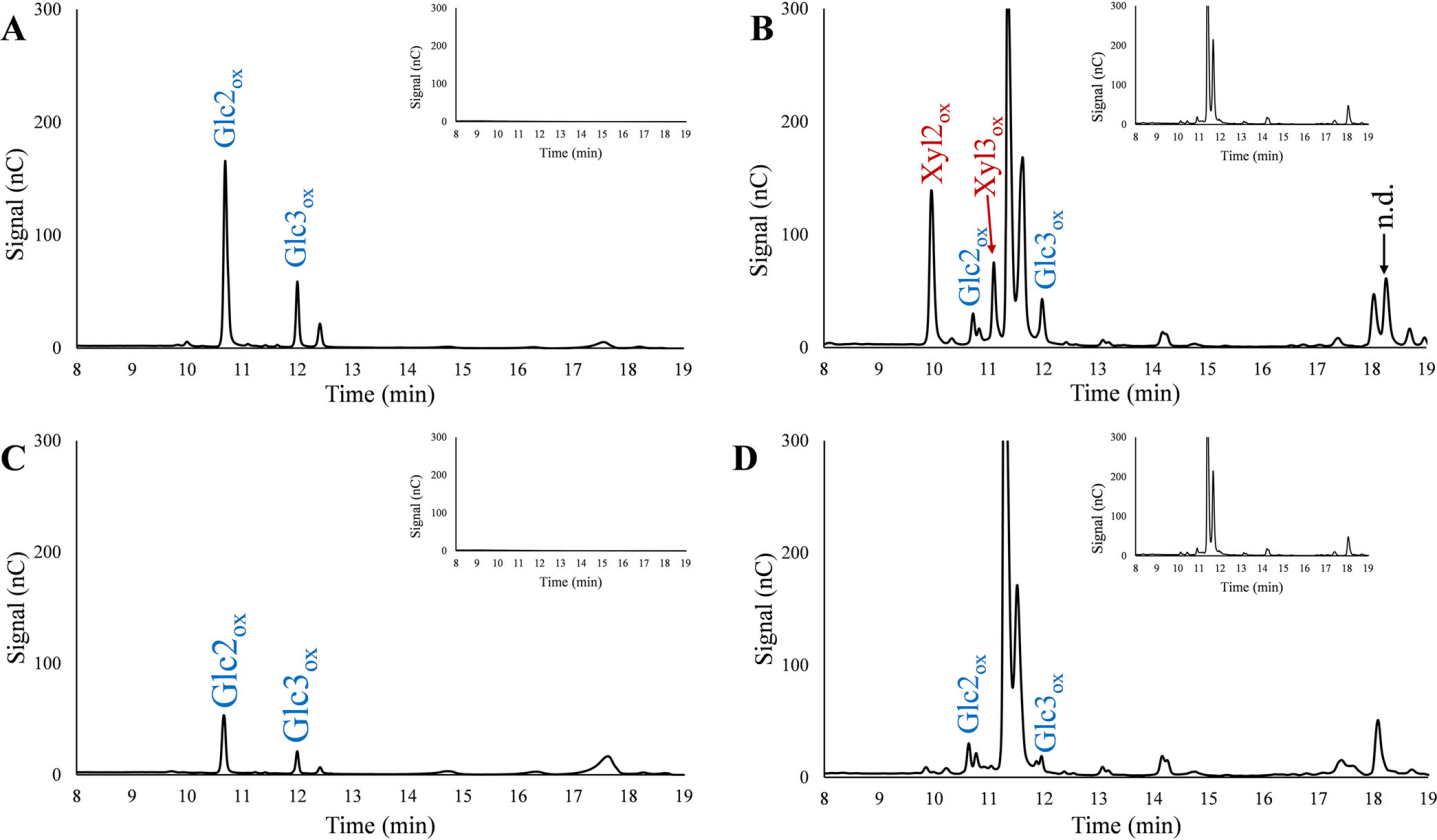
Enzymatic hydrolysis of LPMO products. (A) *Nc*LPMO9F with PASC; (B) *Nc*LPMO9F with PASC and BeWX; (C) *Gt*LPMO9B with PASC; (D) *Gt*LPMO9B with PASC and BeWX. Reductant-free control reactions are shown in the insets. Note the peak marked n.d. (not determined) at 18.4 min, which only appears in reactions with *Nc*LPMO9F and PASC plus BeWX; this could be oxidized glucuronosylated xylan fragments. The two large peaks eluting between 11 and 12 min (independent of the presence of reductant; see the insets in panels B and D) are likely native substituted xylo-oligosaccharides generated from soluble BeWX fragments by *Cj*Xyn10A (see the text and Fig. S3). Glc2_ox_, GlcGlc1A; Glc3_ox_, Glc2Glc1A; Xyl2_ox_, XylXyl1A; Xyl3_ox_, Xyl2Xyl1A.

Next, we hydrolyzed the cello- and xylo-oligosaccharides solubilized by *Nc*LPMO9F when acting on a PASC–BeWX mixture with *Tr*Cel7A and *Cj*Xyn10A in an attempt to quantify LPMO activity on cellulose and xylan, using HPAEC-PAD for quantification of the resulting short, oxidized oligomers. As expected, the resulting product mixtures contained cellobionic acid (GlcGlc1A) and cellotrionic acid (Glc2Glc1A), resulting from oxidation of cellulose (Fig. 5A), as well as xylobionic acid (XylXyl1A) and xylotrionic acid (Xyl2Xyl1A), resulting from oxidation of xylan (Fig. 5B). Next to generating oxidized nonsubstituted xylo-oligomers with DP2 to -3, *Cj*Xyn10A-treated sample contained unknown products, which, considering that the BeWX substrate contained GlcAOMe substitutions, could be native or oxidized glucuronylated xylan fragments (Fig. 5B). The product mixtures obtained upon *Cj*Xyn10A treatment of reactions with BeWX showed large peaks, eluting between 11 and 12 min, independent of the presence of reductant during the LPMO reaction (Fig. 5B and D). Additional treatment of these samples with an α-glucuronidase led to a notable peak shift, indicating that these peaks represent native GlcAOMe-substituted xylooligomers that are liberated from BeWX by *Cj*Xyn10A (Fig. S3). Interestingly, a control experiment with *Gt*LPMO9B showed no xylan oxidation, while the presence of xylan decreased product release from cellulose, indicating that the xylan limits access to the PASC substrate (Fig. 5C and D).

**FIG 6:**
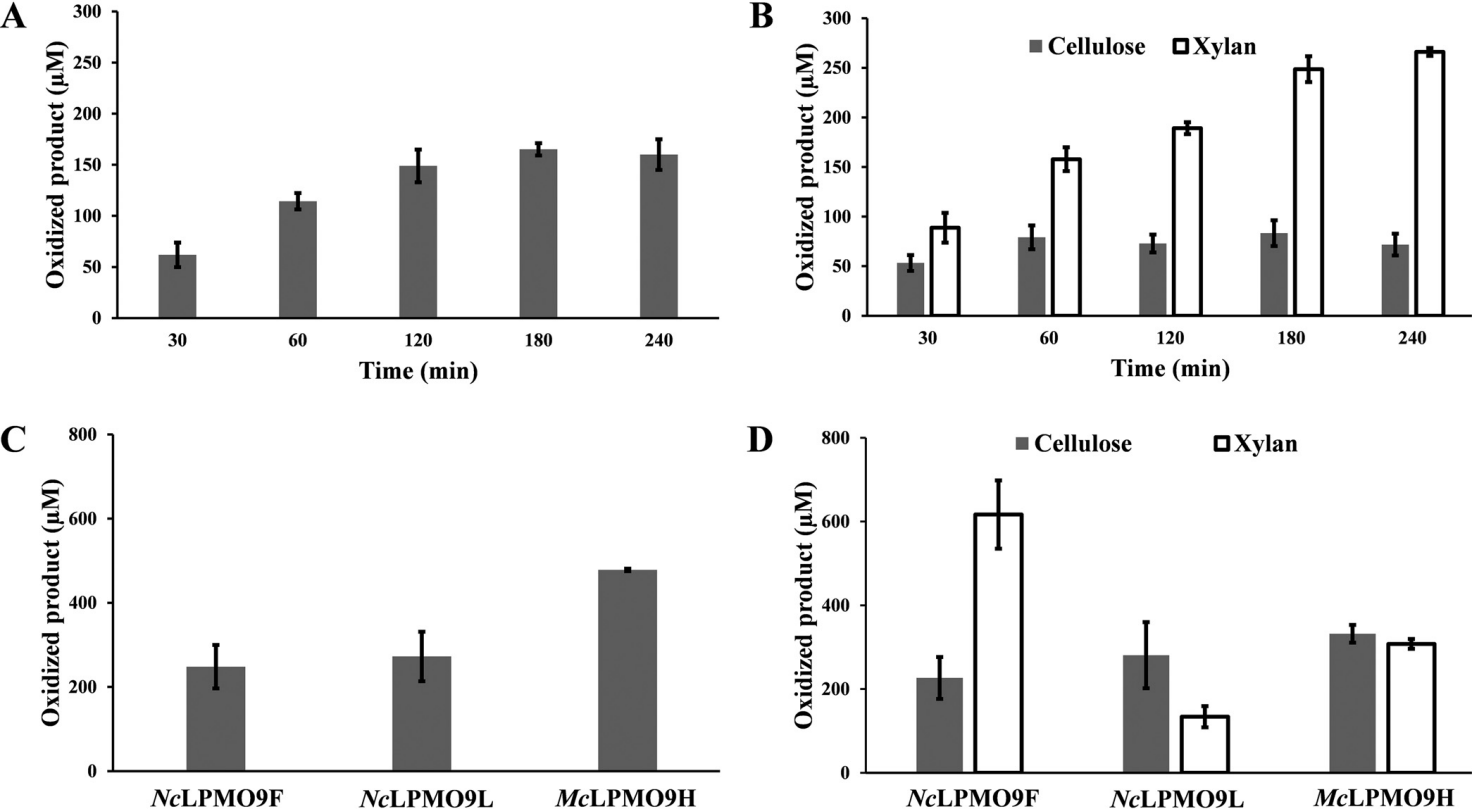
Quantification of oxidized cellulose- and xylan-derived products in reactions with *Nc*LPMO9F. Panels A and B show the formation of oxidized product by *Nc*LPMO9F during a 4-h reaction with PASC or PASC and BeWX, respectively. Panels C and D show oxidized products formed by *Nc*LPMO9F, *Nc*LPMO9L, or *Mc*LPMO9H after 24 h in reactions with PASC or PASC and BeWX, respectively. All reactions were performed with 1 μM LPMO, 1 mM ascorbic acid (AscA), and either 0.4% PASC or 0.4% PASC plus 0.4% BeWX, in 50 mM BisTris-HCl buffer, pH 6.0, at 45°C and 1,000 rpm. Control reactions were performed by replacing AscA with water, and the resulting product mixtures did not show oxidized species (not shown). Before product quantification, product mixtures were hydrolyzed with 1 μM *Tr*Cel7A and 1 μM *Cj*Xyn10A in 75 mM sodium acetate buffer, pH 4.75, for 24 h. Xylobionic acid (XylXyl1A), xylotrionic acid (Xyl2Xyl1A), cellobionic acid (GlcGlc1A), and cellotrionic acid (Glc2Glc1A) concentrations then were measured using HPAEC-PAD and appropriate standards, and the amounts of oxidized DP2 and DP3 products were summed to reach final product levels. Note that for xylan-derived oxidized products, we were only able to quantify linear nonsubstituted products (XylXyl1A and Xyl2Xyl1A). After hydrolysis of LPMO products, we observed a small peak eluting at 18.4 min (Fig. 5) in reactions with PASC and BeWX that likely contains a GlcAOMe-substituted xylan fragment. Thus, the total amount of oxidized xylan-derived product likely is underestimated. Reactions with BeWX only did not yield any oxidized products (not shown). All reactions were performed in triplicate, and standard deviations are indicated.

Quantification of the emergence of oxidized cello- and xylo-oligosaccharides over time in reactions with *Nc*LPMO9F and PASC (Fig. 6A) or the PASC–BeWX mixture (Fig. 6B) revealed that in the presence of AscA, the accumulation of oxidized cello-oligosaccharides stopped after 120 to 180 min, reaching a concentration of 165 μM oxidized products. This product level is far below the theoretical maximum, which is equivalent to the AscA concentration of 1 mM (as also illustrated by the much higher product levels shown in Fig. 6C, discussed below). This low level and the shape of the progress curve indicate that the LPMO lost activity as the reaction progressed. In the reaction mixtures containing both PASC and BeWX, the concentration of oxidized cellulose-derived products was lower, reaching a maximum of 80 μM within 60 min, whereas the concentration of oxidized xylan-derived products (excluding the oxidized glucuronylated xylo-oligosaccharides) was much higher, reaching 266 μM after 240 min. Importantly, in this case, the shape of the progress curve suggests that the reaction proceeded for the full 240-min reaction time.

The progress curves of Fig. 6A and B show some important features. First, the presence of xylan inhibits cellulose conversion by *Nc*LPMO9F, which suggests that BeWX is coating the PASC fibers, making these partially inaccessible to the enzyme. The initial burst in activity on PASC suggests the presence of a more easily accessible cellulose fraction that is not coated by BeWX. Kabel et al. (26) have observed that the degree of substitution of the xylan polymer directly influences the adsorption to cellulose, with unsubstituted xylan having the highest degree of adsorption. Recent data indicate that glucuronoxylan in secondary plant cell walls contains regions with either even or random distribution of glucuronylation (46) and that even distribution favors adsorption to cellulose (27, 28). Hence, it is possible that a variation in distribution of GlcAOMe substitutions along the BeWX polymer yields domains that adsorb to cellulose poorer (domains with more or random substitutions) or better (domains with less or even substitutions), eventually resulting in uneven coating of cellulose in the PASC substrate.

Importantly, the higher levels of xylan-derived products, relative both to cellulose-derived products in the same reaction (Fig. 6B) and to cellulose-derived products in the PASC only reaction (Fig. 6A), clearly show that BeWX, when mixed with cellulose, is a better substrate for *Nc*LPMO9F than PASC. This is also supported by the apparent differences in LPMO stability, which is known to be compromised when the LPMO is provided with reducing equivalents in the absence of sufficient amounts of a suitable substrate (47). The lower apparent stability of the LPMO in the reaction with PASC only and the higher stability in the reaction with BeWX support the notion that xylan is the better substrate.

After establishing that *Nc*LPMO9F generated quantifiable amounts of oxidized xylo-oligosaccharides, we expanded the quantification to include *Nc*LPMO9L and *Mc*LPMO9H. Reactions were set up as described above, with sample collection after 24 h. In the reaction mixtures with PASC only (Fig. 6C), the oxidized product concentration reached similar levels for *Nc*LPMO9F and *Nc*LPMO9L, with 248 and 272 μM, respectively, whereas *Mc*LPMO9H released more oxidized products, reaching a final concentration of 478 μM. No oxidized cello-oligomers were detected in the absence of reductant or LPMO. In reactions with the PASC–BeWX mixture, all three LPMOs generated oxidized xylo-oligosaccharides (Fig. 6D). In line with conclusions drawn from Fig. 3, *Nc*LPMO9F was by far the most xylan-active of the three LPMOs on BeWX. For this enzyme, the apparent ratio of BeWX and PASC oxidization was 2.7:1. *Mc*LPMO9H showed lower xylanolytic activity, and the apparent ratio of BeWX and PASC oxidization was 0.9:1. *Nc*LPMO9L showed even lower xylanolytic activity, and its apparent ratio of BeWX and PASC oxidation was 0.5:1.

### Phylogenetic and structural analysis of xylan-active AA9 LPMOs.

To broaden our understanding of what features could be responsible for the observed activity on xylan, we performed phylogenetic and sequence analyses of *Nc*LPMO9F and *Mc*LPMO9H with 91 uncharacterized homologous proteins selected from a BLAST analysis of the two sequences against the Reference Sequence (RefSeq) database, the remaining 13 *Nc*LPMO9s, and 34 AA9 LPMOs that had been previously characterized to various extents (note that in almost all cases activity on cellulose–xylan mixtures had not been assessed). These analyses (Fig. 1) showed that *Nc*LPMO9F is part of a distinct clade that includes *Mt*LPMO9A, for which (weak) xylanolytic activity was detected (21), and, interestingly, the previously characterized C-1-oxidizing *Tt*LPMO9E from *Thielavia terrestris* (UniProt ID G2RGE5 [48]), for which xylanolytic activity has not yet been assessed. The xylan-active *Mc*LPMO9H and *Nc*LPMO9L occur in closely related sister clades that are separated from the *Nc*LPMO9F clade. Of note, *Mc*LPMO9H is closely related to the previously characterized C-1-oxidizing cellulose-active *Pc*LPMO9D from Phanerochaete chrysosporium (49), for which xylanolytic activity has not yet been assessed.

**FIG 7:**
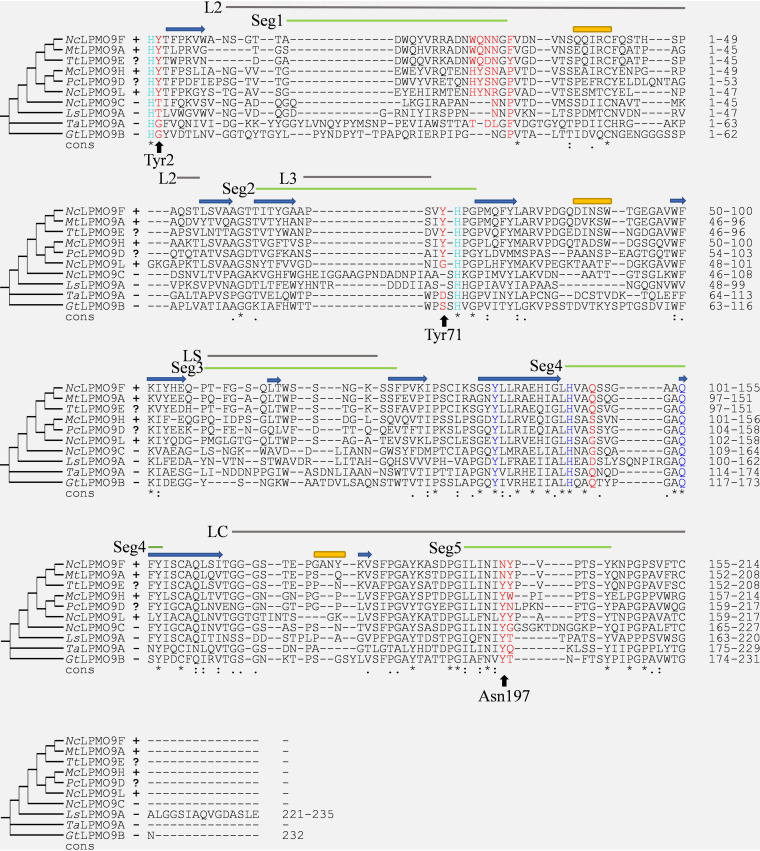
Multiple-sequence alignment of xylan-active and non-xylan-active LPMOs. Expresso alignment of confirmed xylan-active LPMOs (*Nc*LPMO9F, *Nc*LPMO9L, *Mc*LPMO9H, and *Mt*LPMO9A, labeled **+**), potentially xylan-active LPMOs for which xylanolytic activity has not been assessed (*Tt*LPMO9E and *Pc*LPMO9D, labeled ?), and LPMOs for which no xylanolytic activity could be detected in this study (*Nc*LPMO9C, *Gt*LPMO9B, *Ta*LPMO9A, and *Ls*LPMO9A, labeled –). The amino acid residues forming the His brace are light blue, while other conserved residues near the copper site appear in dark blue. Residues that are potentially relevant for xylanolytic activity, as discussed in the text and shown in Fig. 8, are colored red, whereas the three residues used to color the phylogenetic tree of Fig. 1 are indicated by arrows with labels. The secondary structural elements for *Nc*LPMO9F are shown in blue (strands) and yellow (helices) per the PDB crystal structure (4QI8) (36). Surface-exposed and putatively substrate-binding segments (Seg) are indicated by labeled green lines according to Laurent et al. (41), whereas variable regions (called loops) are indicated by labeled gray lines according to Wu et al. (53) and Borisova et al. (59).

The availability of at least one crystal structure for each of the clades with xylan-active LPMOs and the availability of crystal structures for LPMOs found not to be active on glucuronoxylan provide an opportunity to assess possible structural determinants of xylanolytic activity. Despite some recent progress (50–52), the structural determinants of LPMO substrate specificity remain largely unknown. The substrate-binding surfaces of LPMOs vary considerably (Fig. S4), which is due to large sequence variation in specific regions of the LPMO that have been designated the L2, L3, LS, and LC loops (53) and, more recently, segments Seg1 to -5 (41, 54) (Fig. 7). Interestingly, the LPMOs with activity on cellulose-associated glucuronoxylan have shorter L2 and L3 loops, corresponding to shorter Seg1 and Seg2 active-site segments (Fig. 7). From computational and experimental studies of LPMO-substrate complexes, it is clear that both hydrogen bonding and aromatic stacking interactions are important for substrate binding (53, 55–57).

**FIG 8:**
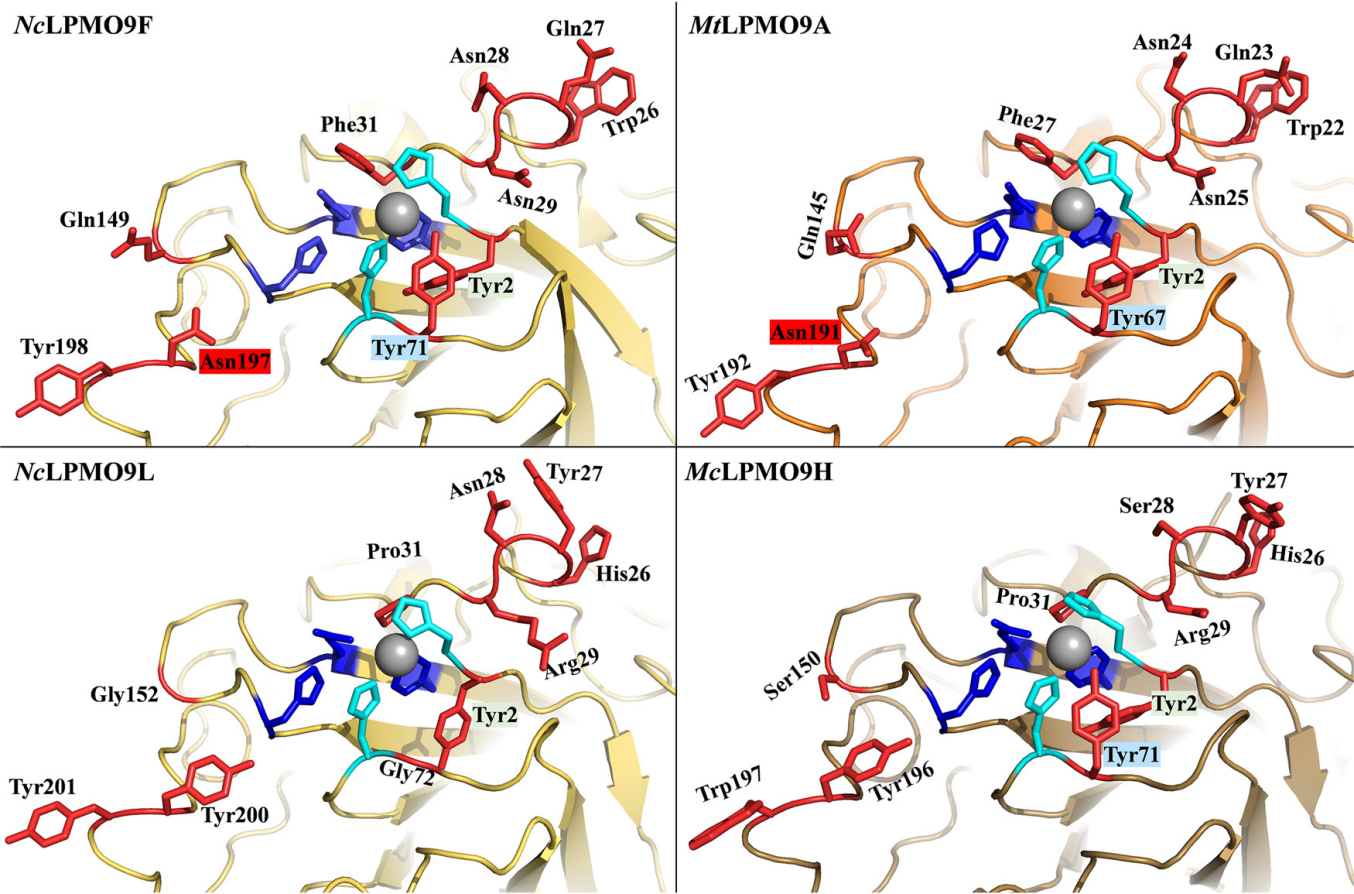
Comparison of the substrate-binding surface of four xylan-active AA9 LPMOs. Surface-exposed residues potentially involved in substrate binding are colored red. The His brace is labeled in light blue, while other conserved residues near the copper site appear in dark blue. Residues that are potentially relevant for xylan binding, as discussed in the text, are colored red. The copper appears as a gray sphere. Labels of residues that were used to color the phylogenetic tree of Fig. 1 are shaded with the corresponding color (Tyr2, Tyr71, and Asn197 in *Nc*LPMO9F). The crystal structure of *Nc*LPMO9F is available from the PDB (entry 4QI8). Models for the remaining LPMOs were built using PHYRE 2.0.

Figures 7 and 8, supported by Fig. S4 to S6, highlight sequence and structural features that seem characteristic for xylan-active LPMOs. Below, we will refer to residue positions according to the position in *Nc*LPMO9F (PDB entry 4QI8). Most notably, all enzymes that cluster with xylan-active LPMOs in Fig. 1 (green cluster) have a conserved tyrosine residue, Tyr2, next to the copper-binding His1 residue (Fig. 7, Fig. S6), which is unique for this subset of AA9 LPMOs. The structures of *Nc*LPMO9F (Fig. 8), *Tt*LPMO9E, and *Pc*LPMO9D show that Tyr2 is not solvent exposed but points inwards and, thus, likely does not contribute directly to substrate binding. Interestingly, in a subset of these LPMOs, the occurrence of this tyrosine is correlated with the occurrence of another, solvent-exposed tyrosine, Tyr71, in *Nc*LPMO9F (see the multiple-sequence alignments [MSAs] in Fig. 7, Fig. S5). In particular, this tyrosine occurs in xylan-active *Mc*LPMO9H and in putatively xylan-active *Pc*LPMO9D and *Tt*LPMO9E but not in xylan-active *Nc*LPMO9L and other AA9 LPMOs, including Tyr2-containing LPMOs (Fig. 1 and 7, Fig. S5). This solvent-exposed Tyr71, which is located within the Seg2 active-site segment, may interact directly with the substrate and may also affect the copper site.

**FIG 9:**
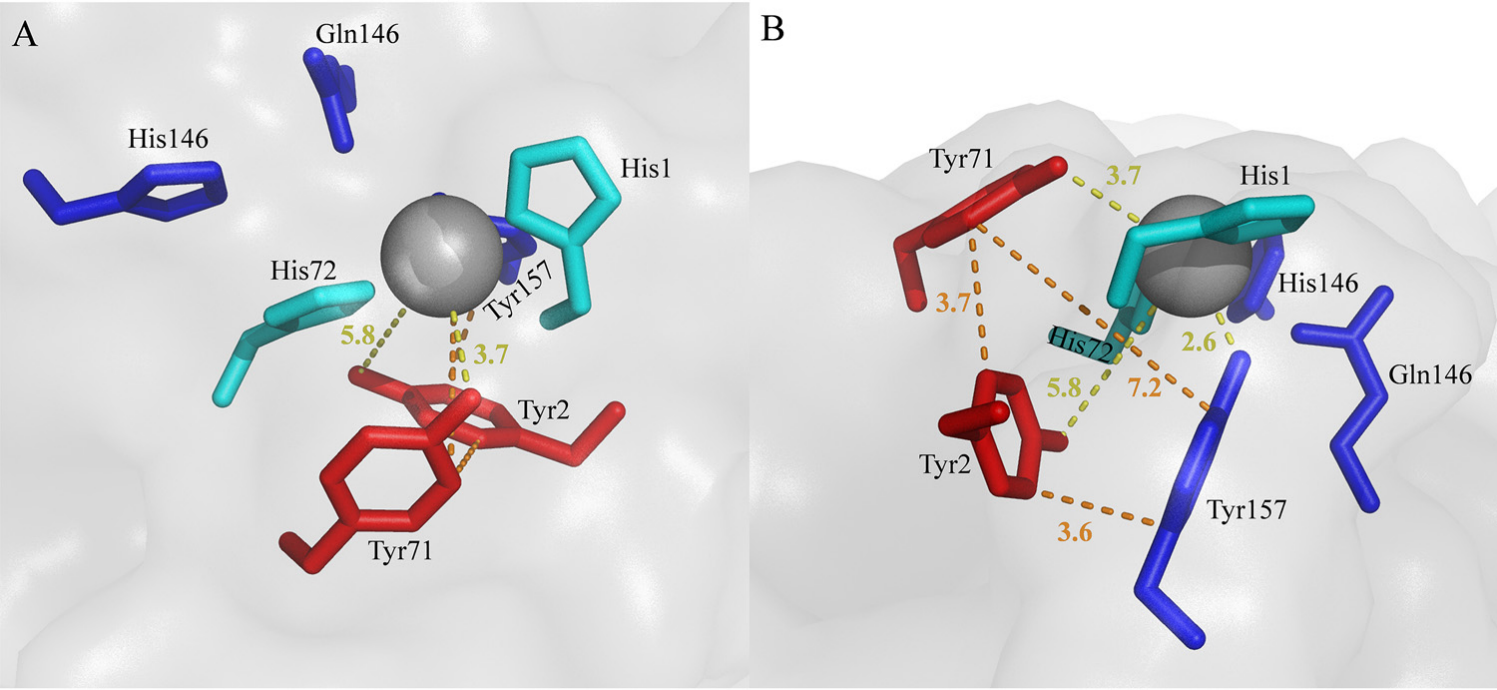
Copper center of *Nc*LPMO9F. The pictures show a top-down view (A) and a side view (B). The closest distances (in Å) between the tyrosine hydroxyls and the copper (yellow lines) and the closest distances between the aromatic rings of the three tyrosines (orange lines) are indicated.

In light of what is known about LPMOs, the occurrence of these two tyrosines (Tyr2 and Tyr71) close to the copper center (Fig. 9) is striking. Both residues are in the second coordination sphere of the copper, and they form a chain of closely connected aromatic residues that also includes the highly conserved tyrosine, Tyr157, in *Nc*LPMO9F whose hydroxyl group occupies the proximal axial copper coordination position. Although further work is needed to elucidate the possible effects of these tyrosines, it is clear that each of them could affect the redox chemistry and redox stability of the copper center (18, 58). The solvent-exposed Tyr71, which has previously been associated with oxidative regioselectivity (59), is of particular interest, since its hydroxyl group occupies a space that is occupied by the C-6 hydroxymethyl group of a glucose in complexes of (C-4-oxidizing) AA9 LPMOs with a cello-oligomer (55, 57). Since there are strong indications from experiments (55, 59) and modeling (60) that cellulose binding to LPMO9s modulates copper site electronics, likely improving oxidant activation, it is tempting to speculate that the hydroxyl group of Tyr71 compensates for the lack of the C-6 hydroxymethyl group in a xylan substrate. While the xylanolytic activity of *Nc*LPMO9L, lacking this tyrosine, shows that Tyr71 is not essential for xylanolytic activity, it is worth noting that of the three xylan-active LPMOs that are compared in Fig. 6D, *Nc*LPMO9L seemed least active on xylan.

Figures 1, 7, and 8 show additional features of the putative substrate-binding surface of xylan-active LPMOs. One particular feature is the presence of an asparagine, Asn197, in *Nc*LPMO9F, in the large majority of members of the *Nc*LPMO9F clade (Fig. S5) at a position where most other AA9 LPMOs, including xylan-active LPMOs outside this clade, have a tyrosine (Fig. 7 and 8, Fig. S4). Computational (53, 61, 62) and experimental studies (61) have shown that this tyrosine interacts with the cellulose substrate through aromatic stacking with the main cellulose chain and hydrogen bonding with adjacent cellulose chains. Figure 8 illustrates that exchanging Tyr with Asn may have a major impact on the substrate-binding surface, and one can speculate that this exchange could explain why *Nc*LPMO9F has the higher activity on xylan (Fig. 6D). Almost all AA9 LPMOs have an exposed aromatic residue in this region, and this is also the case for members of the *Nc*LPMO9F clade (e.g., Tyr198 in *Nc*LPMO9F) (Fig. 8). Interestingly, xylan-active *Nc*LPMO9L and *Mc*LPMO9H have two well-aligned surface-exposed aromatic residues in this region (e.g., Tyr200 and Tyr201 in *Nc*LPMO9L) (Fig. 8), which is an uncommon arrangement among other AA9 LPMOs (Fig. S6). Finally, Fig. 8 illustrates the 26 to 31 region, containing several solvent-exposed residues with hydrogen-bonding potential and showing considerable sequence variation (Fig. S4 and S5), which could affect xylanolytic activity.

### Concluding remarks.

The abundance of LPMO genes in fungal genomes raises interesting questions on their functional variation. The genomes of dikaryotic fungi with a minimum of one AA9 gene contain, on average, 12 AA9 genes, with some species having more than 50 (8). Such multiplicity likely reflects an evolutionary response to the heterogeneity of lignocellulosic substrates. Xylan is the third most abundant biopolymer on earth and is found in the cell walls of all grasses, hardwoods, and softwoods, being an important structural component that coats the cellulose microfibrils and facilitates interactions with lignin (63). Enzymes that remove and depolymerize recalcitrant xylan not only provide the organism with sugars for primary metabolism but also give access to the cellulose underneath. While xylanolytic activity has been detected in the AA14 family (30), this LPMO family is not abundant and is missing completely in 36% of basidiomycete genomes, 76% of ascomycete genomes, and in N. crassa (33). On the other hand, AA9s are more abundant and equally common in ascomycetes and basidiomycetes (8). Considering the abundance of xylan and its presence in insoluble copolymeric structures with cellulose, it would not be surprising if xylanolytic activity was more widespread in the AA9 family than previously thought.

In this work, we demonstrate that *Nc*LPMO9F, known to be cellulose active, and the previously uncharacterized *Nc*LPMO9L are both able to oxidize glucuronoxylan, an important component of grass and hardwood cell walls (64). Importantly, we provide quantitative data for xylan conversion showing that xylanolytic activity is not a weak side activity but rather the primary activity of at least some xylan-active LPMOs. The various ratios between cellulose- and xylan-derived oxidized products for the three xylan-active LPMOs (Fig. 6D) are remarkable and suggest functional variation that may relate, for example, to xylan variability. Xylans come in many forms, showing compositional and structural variation, and it would be of major interest to assess LPMO activity on a wider range of xylan substrates, such as glucuronoarabinoxylan or arabinoxylan.

The discovery that a previously well-characterized LPMO such as *Nc*LPMO9F acts more efficiently on xylan than on cellulose raises the question of whether other well-characterized cellulose-active LPMOs could have undetected capabilities. Side activities or true bifunctionality may have remained undetected because alternative substrates were not tested, because reaction conditions were wrong (e.g., conditions leading to rapid LPMO inactivation), or because alternative substrates were tested alone rather than in combination with cellulose. The latter is not only important for detecting xylanolytic activity (21, 23) but also may be needed to detect activity on other hemicelluloses, such as xyloglucan (65).

The combination of the functional data obtained in this study and the wealth of sequence and structural data for AA9s allowed us not only to point at a cluster of LPMOs that are likely xylan active (Fig. 1) but also to point at structural features near the copper site and on the substrate-binding surface that may be unique or typical for xylan-active AA9s. It is likely that the ancestral LPMO of the xylan-active cluster is of ancient origin, as LPMOs belonging to this cluster occur in both ascomycete and basidiomycete fungal species. It will be exciting to see whether the importance of structural features identified here will be confirmed by future mutagenesis studies of xylan-active and other LPMOs. In this respect, it must be noted that available data indicate that the substrate specificity of LPMOs is a complex trait that depends on multiple residues on and near the substrate-binding surface (50, 51, 66, 67).

The current findings open up several questions that warrant further research. For example, it remains to be seen if the natural function of *Nc*LPMO9F is to degrade (glucurono)xylan or whether it is a truly bifunctional enzyme that has evolved to sequentially oxidize the xylan coating cellulose fibers in natural substrates, followed by oxidation of cellulose. Of note, bifunctional enzymes are not uncommon in cellulolytic enzyme machineries, as exemplified by the particularly powerful *Tr*Cel7B that acts on both cellulose and xylan (68). Such bifunctional enzymes could give a fitness advantage, as production and secretion of enzymes come at a cost for the organism. Another key question for further studies is whether these xylan-active LPMOs could offer advantages in the industrial processing of lignocellulosic biomass. Depending on the feedstock and the pretreatment method used, recalcitrant xylan may be an obstacle for cellulose saccharification, and it is conceivable that LPMOs such as *Nc*LPMO9F can remove this obstacle.

## MATERIALS AND METHODS

### Enzymes.

*Gt*LPMO9B (UniProt ID S7RK00) from *G. trabeum* was produced and purified as described by Hegnar et al. (43). *Mc*LPMO9H (GenBank ID QDV60872.1) from *M. cinnamomea* was produced and purified as described by Hüttner et al. (23). *Nc*LPMO9C (NCU02916; UniProt ID Q7SHI8) and *Nc*LPMO9F (NCU03328; UniProt ID Q1K4Q1) from N. crassa were produced and purified as outlined by Kittl et al. (35). CelS2 (*Sc*LPMO10C) from S. coelicolor was produced and purified as described by Forsberg et al. (69). *Ta*LPMO9A from *T. aurantiacus* (UniProt ID G3XAP7) was produced and purified as reported earlier (70). *Ls*LPMO9A from *L. similis* (GenBank ID ALN96977.1) was produced and purified as described by Rieder et al. (71). Cellobiohydrolase *Tr*Cel7A from Trichoderma reesei (UniProt ID P62694) was prepared from a culture filtrate of *T. reesei* QM 9414 (D-74075; VTT Culture Collection, Finland) as described in reference 72, and the endoxylanase *Cj*Xyn10A from Cellvibrio japonicus (UniProt ID P14768) was purchased from NZYTech (Lisbon, Portugal). β-Xylosidase from Bacillus pumilus and α-glucuronidase from Geobacillus stearothermophilus were purchase from Megazyme.

The coding sequence of *Nc*LPMO9L (gene ID NUC02344; UniProt ID Q7S411), including the native signal peptide, was codon optimized and synthesized between an EcoRI site and a Kozak sequence at the 5′ end (GAATTCGAAAGC) and a stop codon and an Acc65I site (TAAGGTACC) at the 3′ end by GenScript (Piscataway, NJ, USA). The gene was excised using restriction digestion and cloned into a linearized pPink-GAP plasmid (73) using ligation. The resulting plasmid was linearized with AflII (New England BioLabs, Ipswich, MA, USA) and transformed into PichiaPink strain 4 (Invitrogen, Life Technologies Corporation AS, Carlsbad, CA, USA) by following the manufacturer’s instructions. The transformant with the highest protein production level was selected following a previously described protocol (73).

For production and purification of *Nc*LPMO9L, first, an overnight culture of the strain expressing *Nc*LPMO9L was grown in 12.5 ml BMGY medium in a 250-ml baffled shake flask at 29°C and 250 rpm. The overnight culture was used to inoculate 500 ml BMGY in a 2-liter baffled shake flask, followed by incubation at 29°C with mixing at 200 rpm. The supernatants were harvested after 72 h, and the cells were removed by centrifugation at 4°C and 1,500 × *g* for 10 min. The culture supernatants were filtered through a 0.2-μm polyethersulfone (PES) membrane and diluted and reconcentrated several times with Milli-Q water and then with 50 mM BisTris-HCl buffer, pH 6.5, using a VivaFlow 200 tangential crossflow concentrator (molecular weight cutoff, MWCO, 10,000; Sartorius Stedim Biotech GmbH, Göttingen, Germany). *Nc*LPMO9L was purified in two steps. First, the concentrated and buffer-exchanged supernatant was loaded onto a 5-ml CM-FF column equilibrated with 50 mM BisTris-HCl buffer, pH 6.5, using 1.5 ml/min flow rate, and eluted with a linear gradient from 0% to 50% 50 mM BisTris-HCl, pH 6.5, 1 M NaCl. The fractions containing *Nc*LPMO9L were pooled and then concentrated and washed with 50 mM BisTris-HCl, pH 6.5, 150 mM NaCl, using VivaSpin centrifugal tubes (MWCO, 10,000; Sartorius Stedim Biotech GmbH). The protein sample was then loaded onto a 120-ml HiLoad 16/600 Superdex column (GE Healthcare Life Sciences, Uppsala, Sweden) equilibrated with 50 mM BisTris-HCl, pH 6.5, 150 mM NaCl, at 1 ml/min flow rate. The fractions containing *Nc*LPMO9L were pooled, concentrated, and washed with 50 mM BisTris-HCl, pH 6.5, using VivaSpin centrifugal tubes (MWCO, 10,000; Sartorius Stedim Biotech Gmbh), followed by sterilization by filtration.

### Substrates.

Phosphoric acid swollen cellulose (PASC) was prepared from Avicel as described previously (74). Beechwood xylan (BeWX) was purchased from Megazyme (product no. P-XYLNBE; Bray, Ireland). According to the supplier, this xylan contains approximately 13% α-(1→2)-linked substitutions with 4-*O*-methylated glucuronic acid (GlcAOMe).

### LPMO reactions.

LPMO activity was assessed in 100- or 150-μl reaction mixtures, containing either 0.4% (wt/vol) PASC, 0.4% (wt/vol) BeWX, or 0.4% (wt/vol) PASC plus 0.4% (wt/vol) BeWX as the substrate. The PASC–BeWX mixtures were prepared by mixing the two substrates in 50 mM BisTris-HCl buffer, pH 6.0, after which the mixtures were left at room temperature for 30 min to allow BeWX to adsorb onto the cellulose surface. All reactions were performed with 1 μM LPMO and 1 mM AscA in 50 mM BisTris-HCl buffer, pH 6.0. Samples were incubated in an Eppendorf ThermoMixer C (Eppendorf, Hamburg, Germany) at 45°C and 1,000 rpm for 24 h. Control reactions were performed in the absence of AscA. Reactions were stopped by boiling for 5 min, and the soluble fraction was separated from the insoluble material by filtration using a 96-well filter plate (Millipore; Darmstadt, Germany) operated with a vacuum manifold. Soluble fractions were subsequently analyzed using HPAEC-PAD and MALDI-TOF MS, as described below. All experiments were performed in triplicate.

For time series, reaction mixtures were set up with 600 μl total volume. Reaction mixtures contained 1 μM LPMO, 1 mM AscA, and either 0.4% (wt/vol) PASC, 0.4% BeWX (wt/vol), or 0.4% (wt/vol) PASC plus 0.4% (wt/vol) BeWX in 50 mM BisTris-HCl buffer, pH 6.0. The reaction mixtures were incubated in an Eppendorf ThermoMixer C (Eppendorf, Hamburg, Germany) at 45°C and 1,000 rpm. Samples (100 μl) were taken at 30, 60, 120, 180, and 240 min, and the reaction was stopped by boiling for 5 min, after which the soluble and insoluble fractions were separated by centrifugation at 11,000 × *g* for 10 min. Control reactions were performed in the absence of AscA. All reactions were performed in triplicate.

For quantification of product formation, the soluble fraction (25 μl) was mixed with 23 μl 150 mM sodium-acetate buffer, pH 4.75, 1 μl *Tr*Cel7A solution (1 μM final concentration), and 1 μl *Cj*Xyn10A solution (1 μM final concentration). The pH was chosen as a compromise between the optimum pHs for *Tr*Cel7A (pH 4.5) and *Cj*Xyn10A (pH 5.0). *Tr*Cel7A converts native and oxidized cello-oligosaccharides to, primarily, native and oxidized dimers, i.e., cellobiose (Glc2), cellobionic acid (GlcGlc1A), or C-4-oxidized cellobiose (Glc4gemGlc), where the occurrence of the latter two depends on the regioselectivity of the LPMO. In addition, minor amounts of glucose and oxidized trimers may be detected. *Cj*Xyn10A converts xylo-oligosaccharides to shorter linear and branched xylo-oligosaccharides, among which native xylobiose (Xyl2) and xylotriose (Xyl3) and their C-1-oxidized forms, xylobionic acid (XylXyl1A) and xylotrionic acid (Xyl2Xyl1A), can be quantified (see below). Soluble products treated in this way were subsequently analyzed using HPAEC-PAD.

### Detection and quantification of oxidized products.

Oxidized products were analyzed using HPAEC-PAD and MALDI-TOF MS. HPAEC-PAD was performed on a Dionex ICS-5000 system (Dionex, Sunnyvale, CA, USA) equipped with a CarboPac PA200 analytical column (3 by 250 mm) and a CarboPac PA200 guard column (3 by 50 mm). The ICS-5000 instrument was operated with 0.1 M NaOH (eluent A) at a column temperature of 30°C and a flow rate of 0.5 ml/min. A multistep 39-min gradient with increasing amounts of eluent B (0.1 M NaOH plus 1 M NaOAc) was used to elute the products. The gradient was linear from 0 to 5.5% B over 4.5 min; convex upward (gradient type 4) from 5.5% to 15% B over 9 min; concave upward (gradient type 8) from 15% to 100% B over 16.5 min; linear from 100% to 0% B over 0.1 min; stable at 0% B (reconditioning) for 8.9 min.

Chromatograms were analyzed using Chromeleon 7.0 software (Thermo Fischer Scientific, Waltham, MA, USA). Identification of native and oxidized cello- and xylo-oligosaccharides was achieved by using corresponding standards with DP2 to -6. The oxidized cello- and xylo-oligosaccharide standards were prepared by treating 0.05 g/liter Xyl2-Xyl6 or 0.05 g/liter Glc2-Glc6 with 1 μM cellobiose dehydrogenase from *Myriococcum thermophilum* (*Mt*CDH; GenBank ID EF492052.3) (36, 75) in 50 mM Na-acetate buffer, pH 5.0, at 40°C for 20 h. Quantitative estimates of C-1-oxidizing LPMO activity on cellulose and xylan were based on quantification of cellobionic acid (GlcGlc1A) and cellotrionic acid (Glc2Glc1A) for cellulose products and of xylobionic acid (XylXyl1A) and xylotrionic acid (Xyl2Xyl1A) for xylan products (after treating the original products with hydrolases, as described above). These single-compound standards were prepared like the DP2 to -6 mix standards described above. All experiments were performed in triplicate. Analyses of AscA-free and LPMO-free control reactions by HPAEC-PAD showed the presence of small amounts of xylobionic acid, xylotrionic acid, and cellotrionic acid (or other compounds with identical retention times). The areas from these peaks were identical in both types of control reactions and were subtracted when calculating final product concentrations.

Analysis by MALDI-TOF MS was performed with an Ultraflex instrument (Bruker Daltonics GmbH, Bremen, Germany) equipped with a nitrogen 337-nm laser beam, in positive reflector mode, as described previously (20). Sample (1 μl) was mixed with 2 μl matrix solution (10 mg/ml 2,5-dihydroxybenzoic acid in 30% acetonitrile and 0.1% trifluoroacetic acid), applied to a MTP384 ground steel target plate (Bruker Daltonics) and air-dried. Data were collected with flexControl 3.4 (Bruker) and analyzed using mMass v5.5.0 (http://www.mmass.org/).

### Sequence and structure analyses.

For sequence and phylogenetic analyses of *Nc*LPMO9F and *Mc*LPMO9H, the 50 sequences that were most similar to either protein were obtained using the NCBI BLAST tool (https://blast.ncbi.nlm.nih.gov/Blast.cgi) searched against the UniProt RefSeq database. The sequences were manually checked, and a total of seven incomplete or duplicate sequences were removed. The sequences of *Tt*LPMO9E (UniProt ID G2RGE5) and *Mt*LPMO9A (UniProt ID G2QNT0) were among the 50 most similar to *Nc*LPMO9F, while no characterized LPMOs were among the top 50 most similar to *Mc*LPMO9H. A multiple-sequence alignment (MSA) was generated using the resulting data set of 93 AA9 sequences, all 14 *Nc*LPMO9s, *Mc*LPMO9H, and a selection of 32 characterized AA9 LPMOs, using only the AA9 domain and leaving out signal peptides. The MSA was made with T-Coffee’s Expresso tool (http://tcoffee.crg.cat/apps/tcoffee/index.html), which takes into account structural information (76), and was processed using ClustalX 2.1 (77). The resulting MSA (containing 140 sequences) was used for phylogenetic analysis using the ProtTest 3.4 software package (78) by calculating likelihood scores using all included substitution matrices, all improvements (+I, +G, +I +G), and 4 categories for rate variation, empirical amino acid frequencies, and a fixed BIONJ JTT tree for base likelihood calculations. A consensus tree was built with all 120 likelihood scores using the Akaike information criterion (AIC). The resulting consensus tree was edited for publication using iTol v5 (https://itol.embl.de/) (79).

Structure analysis was performed using PyMOL 0.99 (80). The following structures were downloaded from the Protein Data Bank (PDB): 2VTC (*Tr*LPMO9B), 2YET (*Ta*LPMO9A), 3EII (*Tt*LPMO9E), 4B5Q (*Pc*LPMO9D), 4D7U (*Nc*LPMO9C), 4EIR (*Nc*LPMO9D), 4EIS (*Nc*LPMO9M), 4QI8 (*Nc*LPMO9F), 5ACF (*Ls*LPMO9A), 5FOH (*Nc*LPMO9A), 5NLT (*Cv*LPMO9A), 5NNS (*Hi*LPMO9B), 5O2W (*Tr*LPMO9A), 5UFV (*Mt*PMO3, or MYCTH_92668), 6H1Z (*Af*LPMO9B from Aspergillus fumigatus), and 6RS6 (*Ls*LPMO9B). Models for *Gt*LPMO9B, *Mc*LPMO9H, *Mt*LPMO9A (MYCTH_85556), and *Nc*LPMO9L were built using the PHYRE2 Protein Fold Recognition Server (81) in the “intensive” modeling mode, using only the AA9 domain and removing the signal peptide. All models and PDB structures were aligned to the crystal structure of *Nc*LPMO9F for structural comparison.
