# Supplementary material for: Quantifying Oxidation of Cellulose-Associated Glucuronoxylan by Two Lytic Polysaccharide Monooxygenases from Neurospora crassa
Source: Appl Environ Microbiol. 2021 Nov 24;87(24):e01652-21. doi: 10.1128/AEM.01652-21 (PMC8612270; doi:10.1128/AEM.01652-21)
Supplement: Supplemental file 1 — Fig. S1 to S6. Download aem.01652-21-s0001.pdf, PDF file, 3.6 MB [file aem.01652-21-s0001.pdf]

Quantifying oxidation of cellulose-associated glucuronoxylan by two lytic polysaccharide monooxygenases from *Neurospora crassa*

Olav A. Hegnar[1], Heidi Østby[1], Dejan M. Petrović[1], Lisbeth Olsson[2,3], Anikó Várnai[1], Vincent G.H. Eijsink[1,#]

[1]Norwegian University of Life Sciences, Faculty of Chemistry, Biotechnology and Food Science, Ås, Norway

[2]Department of Biology and Biological Engineering, Division of Industrial Biotechnology, Chalmers University of Technology, Gothenburg, Sweden

[3]Wallenberg Wood Science Center, Chalmers University of Technology, Gothenburg, Sweden

Contents:

Figure S1. Phylogenetic relationship and sequence identities of selected AA9 LPMOs.

Figure S2. HPAEC-PAD chromatograms of product mixtures from reactions with BeWX or PASC and BeWX, in the presence of H_2O_2.

Figure S3. HPAEC-PAD chromatograms of LPMO products treated with different enzymes.

Figure S4. Substrate-binding surface of AA9 LPMOs.

Figure S5. Expresso (T-Coffee) multiple sequence alignments of 41 LPMOs in the *Nc*LPMO9F clade.

Figure S6. Structure-based multiple sequence alignment of 15 AA9 LPMOs with known crystal structures.

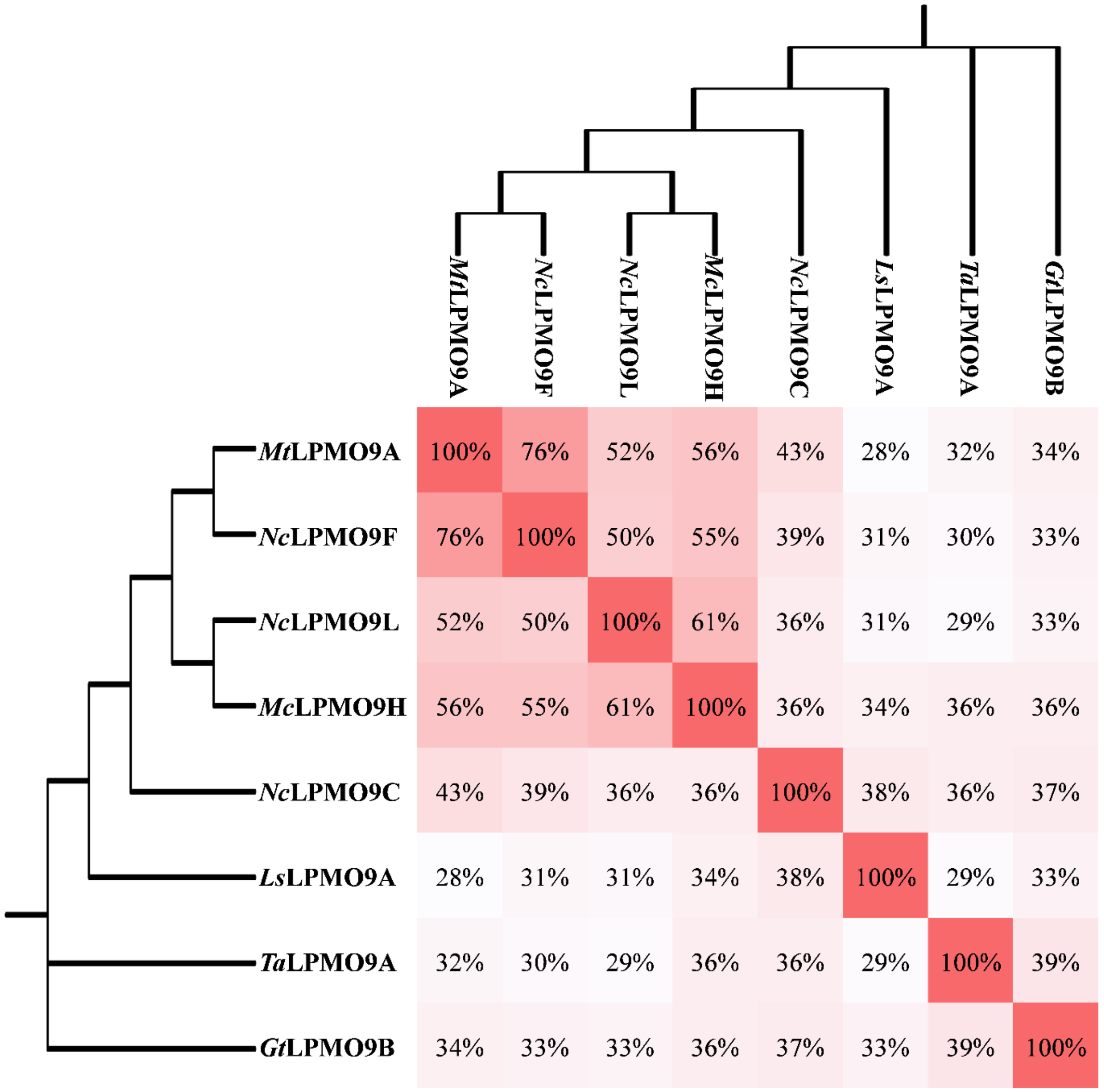

	*Mt*LPMO9A	*Nc*LPMO9F	*Nc*LPMO9L	*Mc*LPMO9H	*Nc*LPMO9C	*Ls*LPMO9A	*Ta*LPMO9A	*Gt*LPMO9B
*Mt*LPMO9A	100%	76%	52%	56%	43%	28%	32%	34%
*Nc*LPMO9F	76%	100%	50%	55%	39%	31%	30%	33%
*Nc*LPMO9L	52%	50%	100%	61%	36%	31%	29%	33%
*Mc*LPMO9H	56%	55%	61%	100%	36%	34%	36%	36%
*Nc*LPMO9C	43%	39%	36%	36%	100%	38%	36%	37%
*Ls*LPMO9A	28%	31%	31%	34%	38%	100%	29%	33%
*Ta*LPMO9A	32%	30%	29%	36%	36%	29%	100%	39%
*Gt*LPMO9B	34%	33%	33%	36%	37%	33%	39%	100%

Figure S1. Phylogenetic relationship and sequence identities of selected AA9 LPMOs. The matrix table shows the sequence identities of *Nc*LPMO9F and *Nc*LPMO9L, previously known xylan-active LPMOs *Mt*LPMO9A and *Mc*LPMO9H, and additional well-characterized LPMOs that were used in the present study. The sequence identities apply to the AA9 catalytic domain only, without signal peptides, linkers and CBMs. The phylogenetic consensus tree was built using ProtTest 3.4 using an Expresso (T-Coffee) MSA of the catalytic domains only.

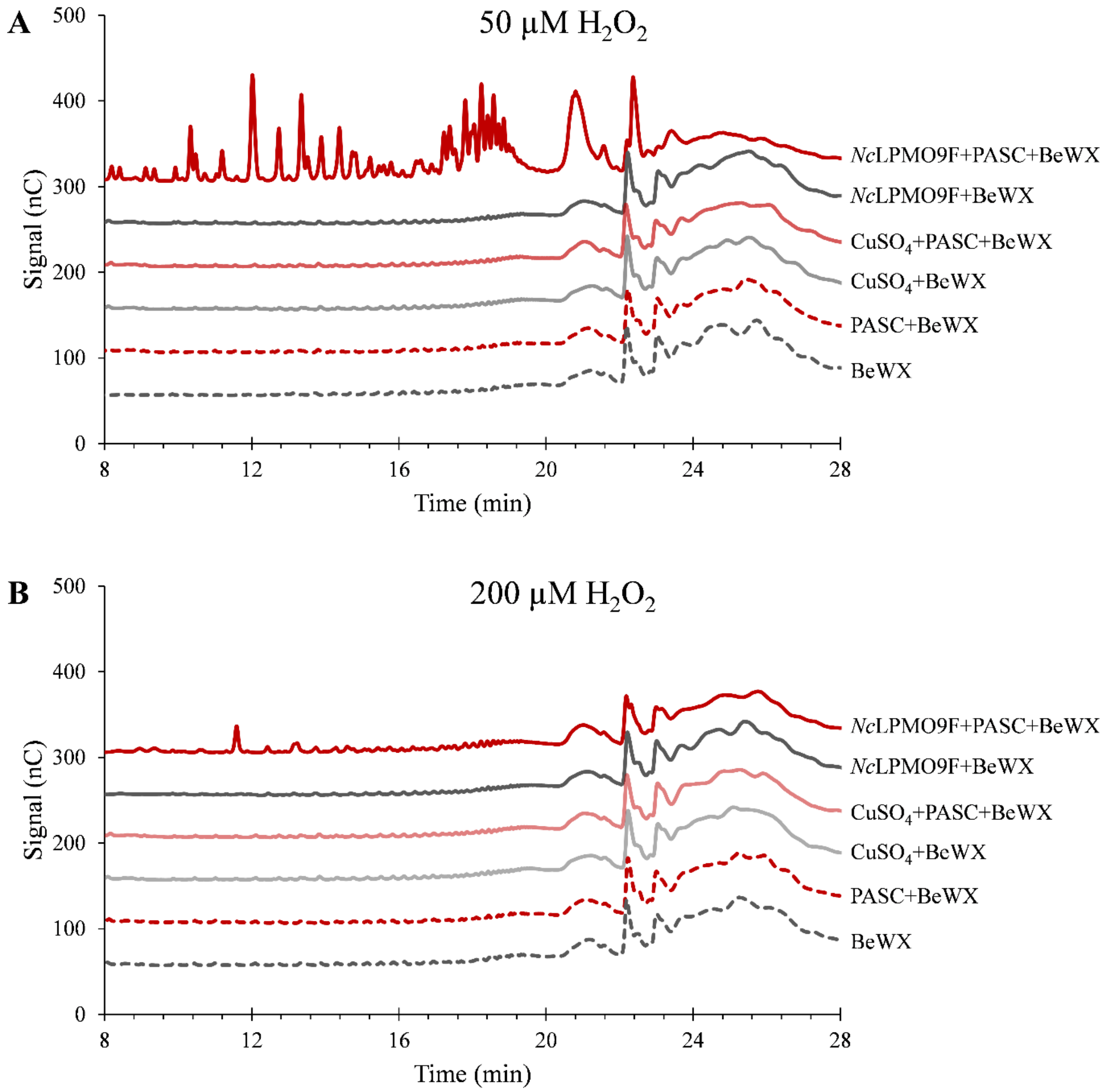

Figure S2. HPAEC-PAD chromatograms of product mixtures from reactions with BeWX or PASC and BeWX, in the presence of H_2O_2. All reactions were performed in 50 mM BisTris-HCl pH 6.0 and were initiated with the addition of 1 mM AscA. Reactions contained either 1 µM *Nc*LPMO9F (solid lines) or 1 µM $CuSO_4$ (transparent lines), or none of these two (dashed lines), and either 0.4% BeWX (w/v) (grey) or 0.4% PASC and 0.4% BeWX (w/v) (red), and either 50 (**A**) or 200 (**B**) µM H_2O_2. . All reactions were incubated at 45°C for 24 hours. All reactions were performed in duplicate and in the duplicate reactions showed similar product profiles in all cases.

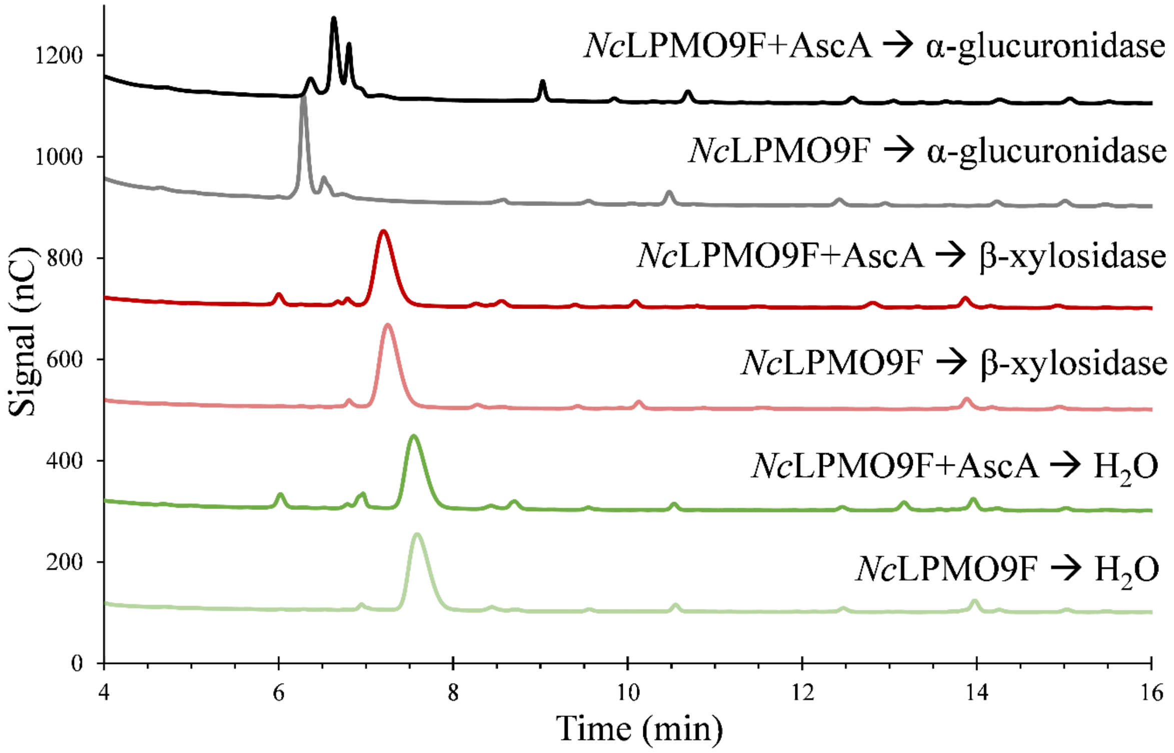

Figure S3. HPAEC-PAD chromatograms of LPMO products treated with different enzymes. A mixture of BeWX (0.4% w/v) and PASC (0.4% w/v) was treated with 1 µM µM *Nc*LPMO9F in the absence or presence of 1 mM AscA, as indicated, in 50 mM BisTris-HCl buffer pH 6.5 at 45°C, overnight. The resulting products where then hydrolyzed with 1 µM *Tr*Cel7A and 1 µM *Cj*Xyn10A for 24 hours in 75 mM sodium acetate buffer pH 4.75 at 37°C**.** Finally, the resulting hydrolysis products were treated with either α-glucuronidase, β-xylosidase or H_2O for 24 hours, as indicated in the chromatograms to the right or the arrows. α-glucuronidase reactions were performed at 70°C in 75 mM Tris buffer pH 7.0, and β-xylosidase reactions were performed at 35°C in 75 mM Tris buffer pH 7.5. Treatment with α-glucuronidase resulted in a clear shift in the large peak at 7.5 minutes in the non- α-glucuronidase treated product mixture, indicating that this peak is composed of GlcAOMe-substituted xylooligomers**.** The effect of xylosidase treatment was less clear, although a minor peak shift may have occurred. All reactions were performed in triplicate and resulted in similar product profiles. The

difference between the elution patterns shown here and those shown in Fig. 5 of the main manuscript are due to differences in the experimental conditions.

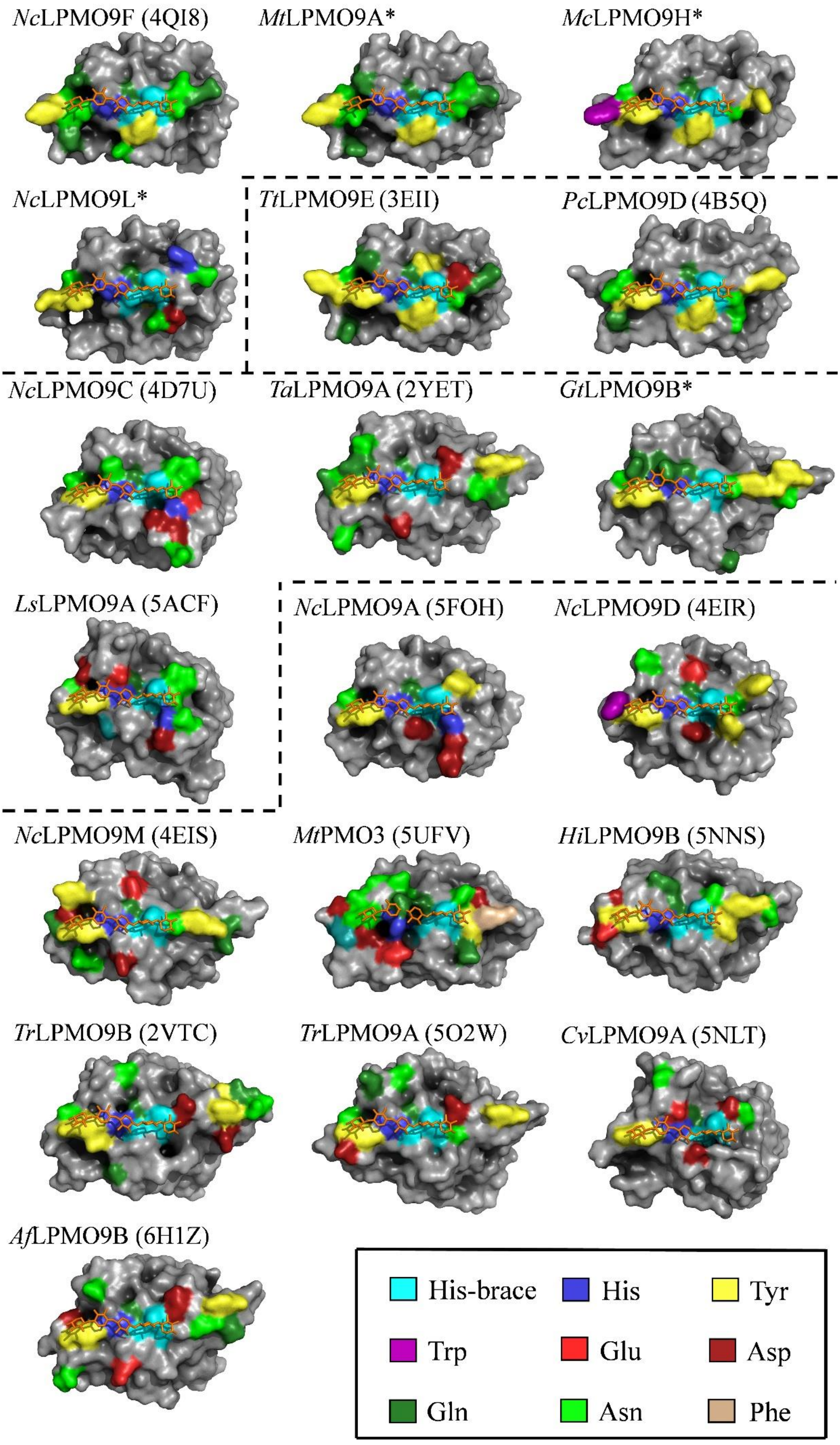
NcLPMO9F (4QI8)
MtLPMO9A*
McLPMO9H*
NcLPMO9L*
TtLPMO9E (3EII)
PcLPMO9D (4B5Q)
NcLPMO9C (4D7U)
TaLPMO9A (2YET)
GtLPMO9B*
LsLPMO9A (5ACF)
NcLPMO9A (5FOH)
NcLPMO9D (4EIR)
NcLPMO9M (4EIS)
MtPMO3 (5UFV)
HiLPMO9B (5NNS)
TrLPMO9B (2VTC)
TrLPMO9A (5O2W)
CvLPMO9A (5NLT)
AfLPMO9B (6H1Z)
His-brace
His
Tyr
Trp
Glu
Asp
Gln
Asn
Phe

Figure S4. Substrate-binding surface of AA9 LPMOs. The figure shows 15 crystal structures labelled with enzyme name and PDB code and PHYRE2 models of *Mt*LPMO9A, *Mc*LPMO9H, *Nc*LPMO9L and *Gt*LPMO9B (marked with *). Residues that are commonly involved in protein-carbohydrate interactions and are located on the substrate-binding surface are coloured, according to the legend in the figure. Note that Tyr2 as discussed in the main text is not solvent-exposed and thus not visible in this view. The dashed lines indicate the following grouping of the structures and models, from top to bottom: LPMOs with proven xylanolytic activity (*Nc*LPMO9F, *Mt*LPMO9A, *Mc*LPMO9H and *Nc*LPMO9L), LPMOs phylogenetically close to LPMOs with proven xylanolytic activity (*Tt*LPMO9E and *Pc*LPMO9D), LPMOs tested in the present study with no detected xylanolytic activity (*Nc*LPMO9C, *Ta*LPMO9A, *Gt*LPMO9B and *Ls*LPMO9A), and nine other AA9 LPMOs. Xylopentaose (Xyl5) is shown in orange and has been superimposed from the crystal structure of *Ls*LPMO9A complexed with Xyl_5 (5NLO) [1].

```
NcLPMO9F            HYTFPKVWANSG-TT-ADWQYVRRADNWQNNGFVDNVNSQQIRCFQ-STHSP---AQS-TLSV
MtLPMO9A            HYTLPRVG-----TG-SDWQHVRRADNWQNNGFVGDVNSEQIRCFQ-ATPAG---AQD-VYTV
TtLPMO9E            HYTWPRVND-----G-ADWQQVRKADNWQDNGYVGDVTSPQIRCFQ-ATPSP---APS-VLNT
KIH88215.1_17-2     HYTFSAL-IEGGQSS-GDWVAVRKTVNWQNNGPVTDVTSNDIRCYE---LSPGTGAAE-TINV
EPE10402.1_17-2     HYTFSSVLVDGK-AT-GDWTAVRKTKNWQNNGPVTDVTSDDIRCFE---LSP-GTAASLTANV
OAA61090.1_18-2     HYTFSALLADAK-NT-GDWIAVRKTKNWQNNGPVTDVTSPDIRCYELDPGTP---ASL-TANV
RKU48715.1_17-2     HYTFPDLVANGQ-PT-GDWVNVIKTTHWQNNGFVGDVTSTGIRC-D-ALQMG---SAK-TYNV
OIW25186.1_17-2     HYTFPQLLAGGK-AT-GDWVNVIKTTHWQNNGFVGDVKSEGIRC-D-ALSMG---SAT-TATV
KAB5511251.1_17     HYTFPQLLAGGK-AT-GEWVNVIKTTHWQNNGFVGDVKSEGIRCDA-LSYGS---A-A-TISV
KAB5543027.1_17     HYTFPQLLAGGK-AT-GDWVNVIKTTHWQNNGFVGDVKSEGIRCDA-LSYGS---A-S-TINV
PKS12653.1_18-2     HYTFPSIQ--S---T-GDWEYVRKADNWQNNGFVGDVSSNQMRCFQ-SREEP---SKA-TLSV
XP_016642286.1      HYTFPSIQ--S---T-GDWQYVRRADNWQSNGFVGSVTSEQMRCFQ-SREEP---SKA-TFTV
XP_006691284.1      HYTFPRVQ--N---G-FDWQYVRRADNFQSNGFVADVNSQQIRCFQ-NIHSP---AQA-TLNV
XP_003346672.1      HYTFPKVWANSG-TT-ADWQYVRRADNWQNNGFVDNVNSNQIRCFQ-SSHSP---AQS-TLSV
XP_009851630.1      HYTFPKVWANSG-TT-ADWQYVRKADNWQNNGFVDNVNSQQIRCFQ-SSHSP---AQS-TLSV
XP_001906810.1      HYTLPRVG--N---G-ADWQHVRRADNWQNNGFVGSVTSPQIRCFQ-NSVAG---ASQ-TYNV
KXX81644.1_18-2     HYTLPKVN-----NG-ADWQHVRRADNWQNNGFVGNVNSEQIRCFQ-SSHAA---APA-VLNV
XP_001225931.1      HYTLPKVG-----SG-SDWQHVRRADNWQNNGFVGDVNSAQIRCFQ-SSSAG---AQD-VYTV
KKY39326.1_17-2     HYTLPSIQ--NS----AAWTAVRQAKNWQDNGFVGNVQSSDIRCNQ--LYA----GNS-TVSV
POS78277.1_17-2     HYTLPSIQ--NS----AAWTVVRQAKNWQDNGFVRDVKSNDIRCNQ--LYA----GNS-SVSV
KAF2443395.1_17     HYTLPTIN---SAPA---WSAVRQAKNWQDNGFVGDVTSSDIRCNQ-L--SA---GNE-TVAV
KAF9732217.1_17     HYTLPTVN---G-AP-A-WSAVRQAKNWQNNGFVGDVTSSDIRCNQ-L--SP---GNE-TVAV
XP_018032121.1      HYTLPTIN---G-AP-A-WSAVRQAKNWQNNGFVGDVTSSDIRCNQ-L--YA---GNE-TVSV
RMZ71950.1_20-2     HYTLPSLN-SD---G-T-WLHVRQAKNWQDNGFVGDVTSSDIRCNQ-LKPGT---S-G-ALSV
KAF7447279.1_18     HYTLPSIN-SD---G-T-WVHVRQAKNWQDNGFVGDVTSSDIRCNQ-LRPGT---S-G-ALSV
KAE8845688.1_18     HYTLPSIN-SD---G-T-WVHVRQAKNWQDNGFVGDVTSSDIRCNQ-LR-SG---TSG-ALSV
OAK94616.1_17-2     HYTLPIIN--SD-SK---WTHIRQAKNWQNNGFVGDVSSSDIRCNQ-L--SA---GTT-TLSV
XP_008024527.1      HYTLPSVN--G---DAA-WSHTRQAKNWQDNGFVGSVTSSDIRCNQ-L--KA---GTS-TLTV
XP_001805107.1      HYTLPIVN---G-DS-A-WTHVRQAKNWQDNGFVGDVTSSAIRCNQ-L--SP---GKS-TLSV
KNG49969.1_17-2     HYTLPSIN--G---D-APWSHVRQAKNWQDNGFVGDVTSNDIRCNQ-L--KA---GTS-TLSV
XP_007718126.1      HYTLPSIN--G---G-VPWSHVRQAKNWQDNGFVGDVTSNDIRCNQ-LKPGT---A---TMTV
XP_007701840.1      HYTLPSIN--G---G-VPWAHVRQAKNWQDNGFVGDVTSNDIRCNQ-LKPGT---A---TMTV
XP_014073816.1      HYTLPSIN--G---G-VPWSHVRQAKNWQDNGFVGDVTSNDIRCNQ-LKPGT---A---TMTV
RYN51948.1_17-2     HYTLPSLN--G---D-AAWSHVRQAKNWQDNGFVGDVTSNDIRCNQ-L--KA---GTS-TLSV
RII23036.1_17-2     HYTLPSLN--G---D-AAWSHVRQAKNWQDNGFVGDVTSNDIRCNQ-L--KA---GTS-TLSV
XP_018384983.1      HYTLPSLN--G---D-AAWSHVRQAKNWQDNGFVGDVTSNDIRCNQ-L--KA---GTS-TLSV
RYN17673.1_17-2     HYTLPSLN--G---D-AAWSHVRQAKNWQDNGFVGDVTSNDIRCNQ-L--KA---GTS-TLSV
XP_028500253.1      HYTLPSVN-GD---S-A-WSHVRQAKNWQDNGFVGDVTSNDIRCNQ-L--KA---GTS-TLSV
RYN30422.1_17-2     HYTLPSVN--G---D-AAWSHVRQAKNWQDNGFVGDVTSNDIRCNQ-L--KA---GTS-TLSV
OWY49194.1_17-2     HYTLPSVN--G---D-AAWSHVRQAKNWQDNGFVGDVTSNDIRCNQ-L--KA---GTS-TLSV
KAB2105098.1_17     HYTLPSVN--G---D-AAWSHVRQAKNWQDNGFVGDVTSNDIRCNQ-L--KA---GTS-TLSV
cons                *                      :: ::*.** * .* *  :**                  .
```

Tyr2

```
NcLPMO9F            AAGTTITYGAAPSVYHPGPMQFYLARVPDGQDINSWTGEGAVWFKIYHEQPTFGSQ-LTWSSN
MtLPMO9A            QAGSTVTYHANPSIYHPGPMQFYLARVPDGQDVKSWTGEGAVWFKVYEEQPQFGAQ-LTWPSN
TtLPMO9E            TAGSTVTYWANPDVYHPGPVQFYMARVPDGEDINSWNGDGAVWFKVYEDHPTFGAQ-LTWPST
KIH88215.1_17-2     TAGGTIAFQANPDIYHPGPLAFYMAKAPSGQTAASFDGAGAVWFKIYEEQPSFGGQTLTWSSN
EPE10402.1_17-2     TAGSSVGFQANPDIYHPGPLAFYMAKAPAGKTAATFDGSGPVWFKIYEEQPTVGGQSLTWSSN
OAA61090.1_18-2     TAGSSVGFQANPDIYHPGPLAFYLAKVPAGQTAASFDGAGAVWFKIYEEQPTFGGQALTWSSN
RKU48715.1_17-2     TAGSSIGFTANPNIYHPGPVQMYMAKVPAGKTAATWDGSGSVWFKVYQEQPKFGSQ-LTWPNN
OIW25186.1_17-2     AAGSTVGFTAAPNIYHPGPVQMYMARVPDGKTAATWDGSGSVWFKIYQEQPGFGAQ-LTWPST
KAB5511251.1_17     AAGSTVGFAASQAIYHPGPVQVYMARVPDGKTAKNWDGSGSVWFKVYQEQPNFGAQ-LTWPST
KAB5543027.1_17     TAGSTVGFAASQGIYHPGPVQMYMARVPDGKTAKNWDGSGSVWFKVYQEQPNFGAQ-LTWPST
PKS12653.1_18-2     AAGSQVTYNALPNVYHPGPMAFYLAKVPDGQSIDTFDGSGDVWFKIYHEQPNFGGQ-LTWPSN
XP_016642286.1      AAGSQITYNALPNVYHPGPMAFYLAKVPEGQTIDTFDGAGDVWFKIYHEQPNFGGQ-LTWPSN
XP_006691284.1      TAGSTVTYYSNQAIFHPGPMSFYMARVPDGQDINSWKGEGAVWFKIYHEQPTFGSS-LKWSSE
XP_003346672.1      AAGTTITYGAAPNVYHPGPMQFYLARVPDGQDINSWTGEGAVWFKIQHEQPQFGQQ-LTWASN
XP_009851630.1      AAGTTITYGAAPSVYHPGPMQFYLARVPDGQDINSWTGEGAVWFKIYHEQPTFGSQ-LTWSSN
XP_001906810.1      SAGSQLTYYVNPNAYHPGPMQFYLARVPDGQDVTRWDGSGAVWFKIYHEQPTFGQQ-LGWPSL
KXX81644.1_18-2     TAGSSVTYSASPNIYHPGPMQFYLARVPDGQDINSWRGDGAVWFKVYHEQPNFGGQ-LSWPSN
XP_001225931.1      SAGSTVKYSANPNIYHPGPMQFYLARVPDGQDVKSWTGDGAVWFKVYHEQPNFGSQ-LTWPSN
KKY39326.1_17-2     AAGGSVSVSIAPNIYHPGPFQSYLAKVPAGQDINTWDPTSAVWFRIYAEQPTFGSQ-LTWTSN
POS78277.1_17-2     AAGSAVTVSIAPNIYHPGPFQSYLAKVPAGQDVNTWDPTSAVWFKIHAEQPTFGSQ-LTWPSN
KAF2443395.1_17     AAGANVKVAVNPNAYHPGPFQSYLAKVPDGQDVNKWDPTGAVWFRIYAEQPKFGSQ-LSWLSA
KAF9732217.1_17     AAGSSVKVNVNPNAYHPGPFQSYLAKVPEGQDVNKWDPTGAVWFRIYAEQPKFGSQ-LTWLNA
XP_018032121.1      AAGSSVKVNVNPNAYHPGPFQSYLAKVPEGQDVNKWDPTGAVWFRIYAEQPKFGSQ-LTWLSA
RMZ71950.1_20-2     AAGSSVKVSVNPSAYHPGPFQSYLAKVPDGQDVNTWDPTGAVWFRIYAEQPKFGSS-LTWLSA
KAF7447279.1_18     AAGSSVKVSVNPNAYHPGPFQSYLAKVPEGQDVNTWDPTGAVWFRIYAEQPKFGSQ-LTWLSA
KAE8845688.1_18     AAGSSVKVSVNPNAYHPGPFQSYLAKVPEGQDVNTWDPTGAVWFRIYAEQPKFGSQ-LTWLSA
OAK94616.1_17-2     AAGASVKVNVDPSAYHPGPFQSYLAKMPEGQDINTWDPTGAVWFRIYAEQPKFGSQ-LTWLSA
XP_008024527.1      AAGSSVKVSVNPSAYHPGPFQSYLARVPDGVDINTWDPTGAVWFRIYAEQPKFGSQ-LTWLGA
XP_001805107.1      AAGSSVKVNVNPNAYHPGPFQSYLAKMPDGQDINTWDPTGAVWFRIYAEQPKFGSQ-LTWLAA
KNG49969.1_17-2     AAGSNVKVSVNPNAYHPGPFQSYLAKVPEGQDINTWDPTGAVWFRIYAEQPKFGGQ-LTW--L
XP_007718126.1      AAGSSVKVSVNPNAYHPGPFQSYLAKVPAGADINTWDPTGAVWFRIYAEQPKFGSQ-LTW--L
XP_007701840.1      AAGSSVKVSVNPNAYHPGPFQSYLAKVPAGADINTWDPTGAVWFRIYAEQPKFGNQ-LTWLAA
XP_014073816.1      AAGSSVKVSVNPNAYHPGPFQSYLAKVPAGADINTWDPTGAVWFRIYAEQPKFGSQ-LTW--L
RYN51948.1_17-2     AAGSSVKVSVNPNAYHPGPFQSYLAKVPEGQDINTWDPTGAVWFRIYAEQPKFGSQ-LTWLSA
RII23036.1_17-2     AAGSSVKVSVNPNAYHPGPFQSYLAKVPEGQDINTWDPTGAVWFRIYAEQPKLGSQ-LTWLSA
XP_018384983.1      AAGSSVKVSVNPNAYHPGPFQSYLAKVPEGQDINTWDPTGAVWFRIYAEQPKFGSQ-LTWLSA
RYN17673.1_17-2     AAGSSVKVSVNPNAYHPGPFQSYLAKVPEGQDINTWDPTGAVWFRIYAEQPKFGSQ-LTWLSA
XP_028500253.1      AAGSNVKVSVNPNAYHPGPFQSYLAKVPEGQDINSWDPTGAVWFRIYAEQPKFGSQ-LTWLSA
RYN30422.1_17-2     AAGSNVKVSVNPNAYHPGPFQSYLAKVPEGQDINTWDPTGAVWFRIYAEQPKFGSQ-LTWLSA
OWY49194.1_17-2     AAGSNVKVSVNPNAYHPGPFQSYLAKVPEGQDINTWDPTGAVWFRIYAEQPKFGSQ-LIWLSA
KAB2105098.1_17     AAGSNVKVSVNPNAYHPGPFQSYLAKVPEGQDINTWDPTGAVWFRIYAEQPKFGSQ-LTWLSA
cons                 **  :        :****.  *:*: * *     :   . ***::  ::* .* . * *
```

Tyr71

```
NcLPMO9F             G-------------------------KS--SFPVKIPSCIKSGSYLLRAEHIGLHVAQSSGAA
MtLPMO9A             G-------------------------KS--SFEVPIPSCIRAGNYLLRAEHIALHVAQSQGGA
TtLPMO9E             G-------------------------KS--SFAVPIPPCIKSGYYLLRAEQIGLHVAQSVGGA
KIH88215.1_17-2      G------------------------LKT---PSVTIPKCLPDGDYLLRIEHIGLHVAQSAGGA
EPE10402.1_17-2      G------------------------LKT---PSVTLPKCLPNGDYLFRIEHIGLHVAQSTGGA
OAA61090.1_18-2      G------------------------LKT---PSVTIPKCLPDGDYLVRIEHIGLHVAQSAGGA
RKU48715.1_17-2      G-------------------------AS--RVTVTVPKCLQNGEYLFRIEHIALHVAQSSGGA
OIW25186.1_17-2      N-------------------------KA--TQSVTIPRCLKSGDYLLRIEHIALHSASSSGGA
KAB5511251.1_17      N-------------------------KATQSFTV--PRCLKSGEYLVRIEHIALHSAGSSGGA
KAB5543027.1_17      N-------------------------KA--TQSVTVPRCLKSGEYLVRIEHIALHSAGSSGGA
PKS12653.1_18-2      GMSDFTIIQFAIPATIKEYTNNGGTGKS--NFAVTIPSCIPAGDYLLRAEHIGLHAASSPGGA
XP_016642286.1_      G-------------------------KS--NFPVTIPACIAPGDYLLRAEHIGLHAAQSPGGA
XP_006691284.1_      G-------------------------KS--AFPVTIPRCIRPGYYLLRAEHIALHSASTPGGA
XP_003346672.1_      G-------------------------KS--SFPVKIPSCIKSGNYLLRAEHIGLHVAQSSGAA
XP_009851630.1_      G-------------------------KS--SFPVKIPSCIKSGSYLLRAEHIGLHVAQSSGAA
XP_001906810.1_      N-------------------------KG--SFPVTIPRCIRSGYYLLRAEHIALHSASSPGGA
KXX81644.1_18-2      ---------------------------------VPIPRCIRSGYYLLRAEHIGLHVAQSSGGA
XP_001225931.1_      G-------------------------KS--SFDVSIPSCIKAGKYLLRAEHIGLHVAQSSGGA
KKY39326.1_17-2      G-------------------------KS--TMSVTIPKCIPAGDYLMRNEHIGLHVAQSEGAA
POS78277.1_17-2      G-------------------------KS--TMSVTIPKCIPAGNYLMRNEHIALHVAQSQGAA
KAF2443395.1_17      ---------------------------A--NYDIKIPACIAPGKYLMRNEHIALHVAQSSGGA
KAF9732217.1_17      ---------------------------A--NYDVKIPPCIAPGRYLMRNEHIALHVAQSPGGA
XP_018032121.1_      ---------------------------A--NYDIKIPSCIAPGKYLMRNEHIGLHVAQSSGGA
RMZ71950.1_20-2      ---------------------------S--TYNIAIPSCLPAGKYLMRNEHIALHTAGTKGGA
KAF7447279.1_18      ---------------------------A--NYNINIPSCIAPGKYLMRNEHIAIHTAGTRGGA
KAE8845688.1_18      ---------------------------A--SYNINIPSCIAPGKYLMRNEHIALHTAGSRGGA
OAK94616.1_17-2      ---------------------------S--NYDIKIPSCIAPGKYLMRNEHIGLHTAQSAGGA
XP_008024527.1_      ---------------------------S--TYDIKLPSCIPAGKYLMRNEHIAIHTASSVGGA
XP_001805107.1_      ---------------------------A--NYDIKIPSCIAPGKYLMRNEHIAIHNAGTKGGA
KNG49969.1_17-2      A-------------------------AS--NYDIKIPQCIAPGKYLMRNEHIAIHTASSRGGA
XP_007718126.1_      A-------------------------AA--NYDIKIPQCIPAGKYLMRNEHIAIHTAGQRGGA
XP_007701840.1_      G-----------------------------TYDIKIPQCIAPGKYLMRNEHIAIHTAGQRGGA
XP_014073816.1_      A-------------------------AS--SYDIKIPQCIAPGKYLMRNEHIAIHTAGSRGGA
RYN51948.1_17-2      ---------------------------A--TYDVKIPQCIAPGKYLMRNEHIAIHTAQSKGGA
RII23036.1_17-2      ---------------------------A--TYDIKIPQCIAPGKYLMRNEHIAIHTAQSKGGA
XP_018384983.1_      ---------------------------A--TYDIKIPQCIAPGKYLMRNEHIAIHTAQSKGGA
RYN17673.1_17-2      ---------------------------A--TYDIKIPQCIAPGKYLMRNEHIAIHTAQSKGGA
XP_028500253.1_      ---------------------------A--NYDIKIPQCIAPGKYLMRNEHIAIHTAQSKGGA
RYN30422.1_17-2      ---------------------------A--NYDIKIPQCIAPGKYLMRNEHIAIHTAQSKGGA
OWY49194.1_17-2      ---------------------------A--NYDIKIPQCIAPGKYLMRNEHIAIHTAQSKGGA
KAB2105098.1_17      ---------------------------A--NYDIKIPQCIAPGKYLMRNEHIAIHTAQSKGGA
cons                                                  :  * *:  * **.* *:*.:* *   *.*

NcLPMO9F             QFYISCAQLSITGGGSTEPGANYKVSFPGAYKASDPGILININYPVPTSYKNPGPSVFTC
MtLPMO9A             QFYISCAQLQVTGGGSTEPS-Q-KVSFPGAYKSTDPGILININYPVPTSYQNPGPAVFRC
TtLPMO9E             QFYISCAQLSVTGGGSTEPP-N-KVAFPGAYSATDPGILINIYYPVPTSYQNPGPAVFSC
KIH88215.1_17-2      QFYISCGQISVSGGGSGSPK-D-LVAFPGAYKATDPGILININYPVPTSYTNPGPAVFTC
EPE10402.1_17-2      QFYISCGQVTVTGGGSKSPT-D-LVAFPGAYKATDPGILININYPVPTSYTNPGPKVFTC
OAA61090.1_18-2      QFYISCGQMTLSGGGSTTPT-D-LVAFPGAYKATDPGILININYPVPTSYTNPGPSVFTC
RKU48715.1_17-2      QFYISCGQISLSGGGNTA-GSP-TVAFPGAYSASDPGILININYPVPTSYTNPGPKVFTC
OIW25186.1_17-2      QFYISCGQISVTGGGSTE-GAP-TVAFPGAYSASDPGILININYPIPTSYVNPGPKVFTC
KAB5511251.1_17      QFYISCGQISVSGGGSTEGGPT--VAFPGAYSASDPGILININYPVPTSYVNPGPKVFSC
KAB5543027.1_17      QFYISCGQISVSGGGSTEGGPT--VAFPGAYSASDPGILININYPVPTSYQNPGPKVFSC
PKS12653.1_18-2      QFYISCAQLTITGGGSTDPP-N-KVAFPGAYKASDPGIQININYPVPTSYQNPGPAVFSC
XP_016642286.1_      QFYISCAQLTITGGGSTDPP-N-KVAFPGAYKASDPGIQININYPVPTSYQNPGPAVFKC
XP_006691284.1_      QFYISCAQLAVTGGGSTDPP-N-KVSFPGAYKANDPGIQINIYYPVPTSYKNPGPSVFSC
XP_003346672.1_      QFYISCAQLSITGGGNTEPS-N-KVSFPGAYKASDPGILININYPVPTSYKNPGPSVFIC
XP_009851630.1_      QFYISCAQLSITGSGSTEPGANYKVSFPGAYKASDPGILININYPVPTSYKNPGPSVFTC
XP_001906810.1_      QFYISCAQIGVTGGGNTEPS-N-KVSFPGAYSASDPGIQININWPIPTSYRNPGPPVFQC
KXX81644.1_18-2      QFYISCAQIGVTGGGNTDPP-N-KVSFPGAYRASDPGIMININWPIPTSYQNPGPPVFSC
XP_001225931.1_      QFYISCAQIEVTGGGSTEPS-N-KVSFPGAYKASDPGILININWPIPTSYQNPGPPVFKC
KKY39326.1_17-2      QFYLSCGQLSVTGGGSTQPQ-N-LAAFPGAYSSKDPGILININYPVPTSYVNPGPATFTC
POS78277.1_17-2      QFYLSCAQLSVTGGGSTQPQ-N-LVAFPGAYSATDPGILININYPVPTSYKNPGPATFTC
KAF2443395.1_17      QFYLSCAQIEVTGGGSKTPT-N-LVAFPGAYKASDPGILINVNYPVPTSYVNPGPATFTC
KAF9732217.1_17      QFYLSCAQIDVVGGGSQTPT-D-LAAFPGAYRASDPGILININYPVPTSYVNPGPATFVC
XP_018032121.1_      QFYLSCAQIEVTGGGSKTPT-N-LAAFPGAYKASDPGILININYPVPTSYVNPGPATFTC
RMZ71950.1_20-2      QFYLSCGQLEVTGGGSKSPS-N-LVAFPGAYSATDPGILININYPIPTSYKNPGPATFTC
KAF7447279.1_18      QFYLSCAQIEVTGGGSKTPT-N-LVAFPGAYSATDPGILININYPIPTSYKNPGPPTFTC
KAE8845688.1_18      QFYLSCAQIEVTGGGSKAPT-N-LVAFPGAYSATDPGILININYPIPTSYKNPGPATFTC
OAK94616.1_17-2      QFYLSCAQLDITGGGSKAPT-N-LVAFPGAYKSSDPGILININYPIPTSYTNPGPPTFTC
XP_008024527.1_      QFYLSCAQLQITGGGSTNPR-D-LVAFPGAYKATDPGILININYPIPTSYVNPGPPTFTC
XP_001805107.1_      QFYLSCAQLEVTGGGSTAPK-N-LVAFPGAYSATDPGILININYPIPTSYKNPGPDTFTC
KNG49969.1_17-2      QFYLSCAQIEVTGGGSTAPR-N-LVAFPGAYSATDPGIMININYPIPTSYVNPGPPTFTC
XP_007718126.1_      QFYLSCAQIEVTGGGSTSPR-N-LVAFPGAYSATDPGILININYPIPTSYVNPGPPTFTC
XP_007701840.1_      QFYLSCAQIEVTGGGSTSPR-N-LVAFPGAYSATDPGILININYPIPTSYVNPGPPTFTC
XP_014073816.1_      QFYLSCAQIEVTGGGSTSPR-N-LVAFPGAYSATDPGILININWPIPTSYVNPGPPTFTC
RYN51948.1_17-2      QFYLSCGQLEVTGGGSKAPS-N-LVAFPGAYKATDPGILININYPIPTSYTNPGPPTFTC
RII23036.1_17-2      QFYLSCGQLEVTGGGSKAPS-N-LVAFPGAYKATDPGILININYPIPTSYTNPGPPTFTC
XP_018384983.1_      QFYLSCGQLEVTGGGSKAPS-N-LVAFPGAYKATDPGILININYPIPTSYTNPGPPTFTC
RYN17673.1_17-2      QFYLSCGQLEVTGGGSKAPS-N-LVAFPGAYKATDPGILININYPIPTSYTNPGPPTFTC
XP_028500253.1_      QFYLSCAQLEVTGGGSKAPS-N-LVAFPGAYKATDPGILININYPIPTSYTNPGPPTFTC
RYN30422.1_17-2      QFYLSCAQLEVTGGGSKAPS-N-LVAFPGAYKATDPGILININYPIPTSYTNPGPPTFTC
OWY49194.1_17-2      QFYLSCAQLEVTGGGSKAPS-N-LVAFPGAYKATDPGILININYPIPTSYTNPGPPTFTC
KAB2105098.1_17      QFYLSCAQLEVTGGGSKAPS-N-LVAFPGAYKATDPGILININYPIPTSYTNPGPPTFTC
cons                 ***:**.*: : *.*.        .:***** :.**** **: :*:**** **** .* *
                                                               ↑
                                                             Asn197
```

Figure S5. Expresso (T-Coffee) multiple sequence alignments of 41 LPMOs in the *Nc*LPMO9F clade. Enzymes with demonstrated xylan activity are highlighted in yellow. Sequence features of xylan active LPMOs that are discussed in the main text are highlighted with red. Three characteristic sequence features of the clade are also highlighted by arrows and labels: Tyr2, Tyr71 and Asn197 (residue numbering according to *Nc*LPMO9F). Conserved residues in the first and second coordination sphere of the copper appear in light and dark blue, as in Fig. S4.

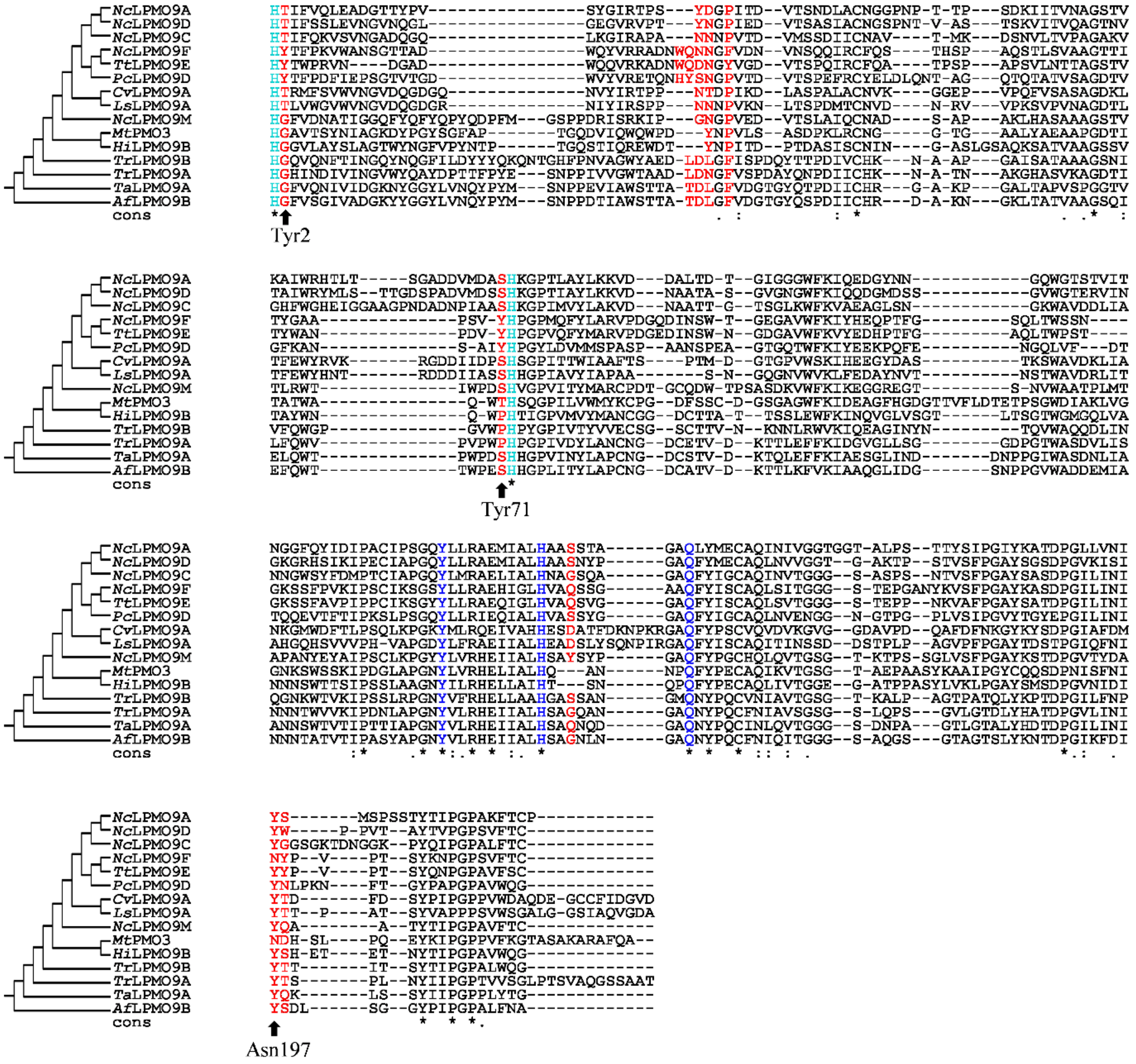

Figure S6. Structure-based multiple sequence alignment of 15 AA9 LPMOs with known crystal structures. Sequence features of xylan-active LPMOs that are discussed in the main text are highlighted with red. The copper-binding histidines appear in light blue, whereas a conserved tyrosine in the proximal axial copper coordination position and a conserved glutamine and histidine in the second coordination sphere appear in dark blue. The PDB codes for the structures are given in Fig. S4 and in the Materials and methods section.

1. Simmons, T.J., et al., *Structural and electronic determinants of lytic polysaccharide monooxygenase reactivity on polysaccharide substrates.* Nature Communications, 2017. **8**(1): p. 1064.
